# RNAi-based modulation of IFN-γ signaling in skin

**DOI:** 10.1016/j.ymthe.2022.04.019

**Published:** 2022-04-27

**Authors:** Qi Tang, Jacquelyn Sousa, Dimas Echeverria, Xueli Fan, Ying-Chao Hsueh, Khashayar Afshari, Nicholas MeHugh, David A. Cooper, Lorenc Vangjeli, Kathryn Monopoli, Ken Okamura, Annabelle Biscans, Adam Clauss, John E. Harris, Anastasia Khvorova

**Affiliations:** 1 RNA Therapeutics Institute, University of Massachusetts Chan Medical School, Worcester, MA 01605, USA; 2 Department of Dermatology, University of Massachusetts Chan Medical School, Worcester, MA 01605, USA; 3 LEO Pharma A/S, Industriparken 55, 2750 Ballerup, Denmark; 4 Immunology and Microbiology Program, University of Massachusetts Chan Medical School, Worcester, MA 01605, USA; 5 Bioinformatics and Computational Biology Program, Worcester Polytechnic Institute, Worcester, MA 01609, USA

**Keywords:** RNAi therapeutics, siRNA delivery, autoimmune disorders, skin immunology, IFN-γ signaling, immunomodulatory drugs, CXCL9/10/11 chemokines, preclinical drug development

## Abstract

Aberrant activation of interferon (IFN)-γ signaling plays a key role in several autoimmune skin diseases, including lupus erythematosus, alopecia areata, vitiligo, and lichen planus. Here, we identify fully chemically modified small interfering RNAs (siRNAs) that silence the ligand binding chain of the IFN-γ receptor (*IFNGR1*), for the modulation of IFN-γ signaling. Conjugating these siRNAs to docosanoic acid (DCA) enables productive delivery to all major skin cell types local to the injection site, with a single dose of injection supporting effective IFNGR1 protein reduction for at least 1 month in mice. In an *ex vivo* model of IFN-γ signaling, DCA-siRNA efficiently inhibits the induction of IFN-γ-inducible chemokines, CXCL9 and CXCL10, in skin biopsies from the injection site. Our data demonstrate that DCA-siRNAs can be engineered for functional gene silencing in skin and establish a path toward siRNA treatment of autoimmune skin diseases.

## Teaser

Dysregulation of IFN-γ signaling has been found in several T cell-mediated autoimmune skin diseases, including lupus erythematosus, alopecia areata, vitiligo, and lichen planus. In this work, Khvorova, Harris, and colleagues developed a therapeutic siRNA that efficiently modulates the IFN-γ signaling to potentiate the treatment of autoimmune skin diseases.

## Introduction

**Figure 1:**
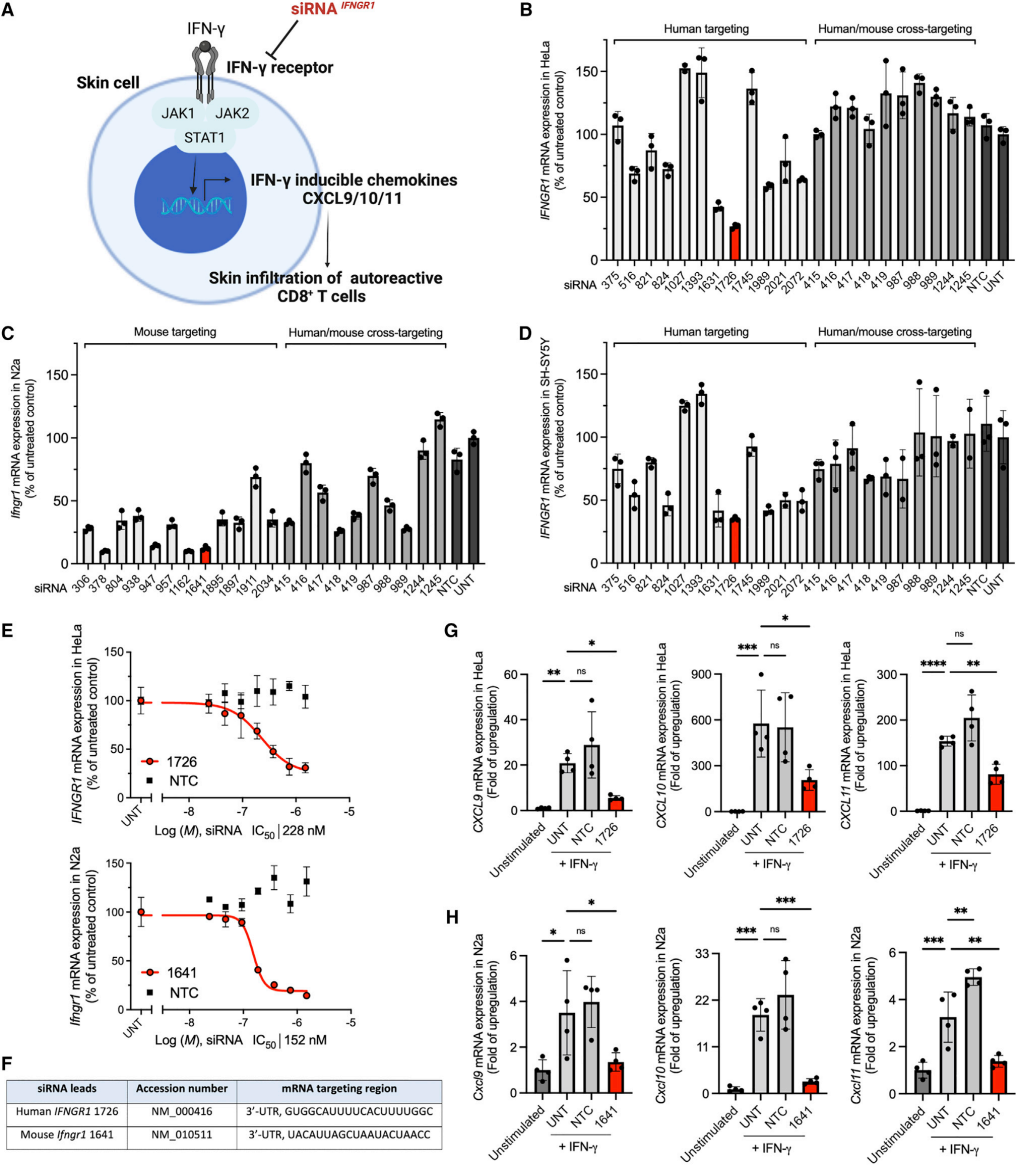
Identification of human *IFNGR1* and mouse *Ifngr1* siRNA leads that efficiently modulate IFN-γ signaling (A) Schematic of siRNA silencing IFN-γ receptor for the modulation of IFN-γ signaling. (B) Human *IFNGR1* silencing in HeLa cells, (C) mouse *Ifngr1* silencing in N2a cells, and (D) human *IFNGR1* silencing in SH-SY5Y cells. Cells were treated with fully modified cholesterol-conjugated siRNAs at 1.5 μM for 72 h. The mRNA levels were measured by using QuantiGene 2.0 assays. siRNA number represents the 5′ position of the mRNA target site. UNT, untreated control. Data are represented as percent of UNT (n = 3, mean ± standard deviation). (E) Dose-response curves of lead siRNA compounds (human 1726, mouse 1641). M, molar concentration of siRNA (n = 3, mean ± standard deviation). (F) Targeting region of lead siRNA in human *IFNGR1* and mouse *Ifngr1* mRNA. (G) Human *CXCL9/10/11*, and (H) Mouse *Cxcl9/10/11* mRNA expressions (QuantiGene 2.0 assay) at 6 h post IFN-γ signaling stimulation; cells were treated with siRNA at 1.5 μM for 72 h before IFN-γ stimulation (n = 4, mean ± standard deviation, one-way ANOVA, ∗p < 0.05, ∗∗p < 0.01, ∗∗∗p < 0.001, ∗∗∗∗p < 0.0001; ns, not significant).

Interferon (IFN)-γ signaling promotes the progression of CD8^+^ T cell-mediated autoimmune skin diseases, such as lupus erythematosus, alopecia areata, vitiligo, and lichen planus.^1^, ^2^, ^3^, ^4^ Autoreactive CD8^+^ T cells in lesional skin produce IFN-γ that binds the IFN-γ receptor to activate the Janus kinase (JAK)-signal transducer and activator of transcription pathway, thereby stimulating the expression of IFN-γ-inducible chemokines, such as CXCL9, CXCL10, and CXCL11. These chemoattractants, in turn, promote the skin infiltration of autoreactive CD8^+^ T cells and worsen autoimmunity (Figure 1A).^5^, ^6^, ^7^

The upregulation of IFN-γ and its related genes in skin biopsies from patients and mouse models of autoimmune skin diseases indicate that targeting the IFN-γ pathway may be an effective therapeutic strategy.^3^^,^^8^^,^^9^ Indeed, the clinical off-label use of small molecule JAK inhibitors (e.g., ruxolitinib, tofacitinib, and baricitinib) has produced promising results in the treatment of alopecia areata and vitiligo.^10^, [11], 12, 13 These medications can be administered systemically or topically. Systemic treatments usually provide better outcomes for severe conditions when large areas of skin are involved, whereas topicals are good for patients with localized symptoms and incur fewer systemic side effects. Topical JAK inhibitors are efficacious in modulating IFN-γ-related autoimmunity; however, JAK inhibition could potentially affect other immune pathways and lead to undesirable activities.^14^ Therefore, we hypothesize that locally targeting IFN-γ receptor, the upstream molecule, may be a viable approach to specifically modulate the IFN-γ signaling in skin.

Small interfering RNAs (siRNAs) are a novel class of drugs that harness endogenous RNA interference (RNAi) to enable specific and sustained modulation of gene expression.^15^^,^^16^ Synthetic siRNAs offer several advantages over small molecule and antibody drugs, including ease of sequence-based design, which allows for rapid drug discovery, and the ability to target disease genes previously considered undruggable.^17^ Currently, conjugate-mediated delivery of siRNAs is the dominant delivery platform in the clinic. The most clinically advanced siRNA conjugate, *N*-acetylgalactosamine (GalNAc), supports selective hepatocyte delivery through asialoglycoprotein receptor uptake and is the basis for multiple approved siRNA drugs.^18^, ^19^, ^20^ GalNAc-conjugated siRNAs show an unprecedented duration of effect (>6 months after a single administration)^21^ driven by compound entrapment in lysosomal and endosomal compartments, generating an intracellular depot of the drug with slow release.^22^ While the usefulness of GalNAc is limited to the liver, hydrophobic conjugates support delivery to extra-hepatic organs, including skin.^23^^,^^24^ Among hydrophobic conjugates, docosanoic acid (DCA) shows promise for safe, multicellular siRNA delivery both locally and systemically.^23^^,^^25^

Here we report the rational development of therapeutic siRNAs that silence the ligand binding chain of the IFN-γ receptor (*IFNGR1*) for the modulation of IFN-γ signaling. We screened a panel of fully chemically modified siRNAs *in vitro* and identified multiple functional hits that enable efficient silencing of both human *IFNGR1* and mouse *Ifngr1* mRNAs. We systematically evaluated hydrophobic conjugates and scaffold valency on siRNA and identified DCA-conjugated siRNA as showing the highest efficacy in skin local to the injection site, with productive delivery to multiple skin cell types. In addition, we found that a single subcutaneous injection of DCA-siRNA supports sustained IFNGR1 protein reduction in skin for at least 1 month. Finally, we demonstrate that DCA-siRNA significantly decreases the induction of IFN-γ-inducible chemokines, CXCL9 and CXCL10, in an *ex vivo* skin model of IFN-γ signaling. These findings highlight RNAi-based gene modulation as a potential localized treatment for autoimmune and other skin diseases.

## Results

### *In vitro* screen identifies lead siRNA compounds that silence human *IFNGR1* and mouse *Ifngr1* mRNAs

To identify active siRNAs that silence human *IFNGR1* and mouse *Ifngr1*, we first performed bioinformatics analysis to predict functional sequences that cover the 5′-untranslated region (UTR), open reading frame, or 3′-UTR regions of target mRNA in both species. A panel of the top 24 species-specific siRNA sequences (12 human and 12 mouse) were selected for *in vitro* screen. Although there is limited homology between human *IFNGR1* and mouse *Ifngr1* mRNAs, we were able to generate an additional 10 human and mouse cross-targeting candidates based on the siRNA design criteria.^26^^,^^27^ In addition to the mRNA homology of species, chemical modifications of nucleotides also highly affect the available sequence space for designing cross-targeting siRNAs.^28^ In this study, all screens used fully chemically modified, asymmetric siRNA scaffolds with optimized 2′-O-methyl (2′-OMe), 2′-fluoro (2′-F) and phosphorothioate (PS) linkages that have been shown to improve stability.^17^^,^^29^ The 3′ end of sense strands were hydrophobically conjugated to cholesterol, which promotes cellular internalization without the need for lipid formulation.^30^ All sequences and chemical modification patterns of siRNAs are summarized in the supplemental information (Tables S1–S3).

In primary screens using Quantigene 2.0 assays, we identified multiple functional siRNA hits with effective silencing up to approximately 75% of human *IFNGR1* and approximately 90% of mouse *Ifngr1* (Figures 1B and 1C). Interestingly, 4 of 10 cross-targeting siRNAs (415, 418, 419, and 989) that showed greater than 50% silencing efficacy in mouse N2a neuroblastoma cells were not effective in human HeLa epithelial cells. To investigate whether human cell type affects *IFNGR1* mRNA accessibility, we re-screened the compounds in human neuroblastoma SH-SY5Y cells. Consistent with the results in HeLa cells, minimal or no efficacy was observed for cross-targeting siRNAs in SH-SY5Y cells (Figure 1D). The median inhibition concentration (IC_50_) values were determined for the top four hits from each primary screen (human 1631, 1726, 1989, and 2072; and mouse 378, 947, 1162, and 1641) using seven-point dose-response curves (Figures 1E and S1). Based on the IC_50_ values, human 1726 (IC_50_ 228 nM) and mouse 1641 (IC_50_ 152 nM) were selected as the lead compounds (Figure 1F). We also treated human HeLa, SY-SH5Y, and mouse N2a cells with the lead compounds and determined IFNGR1 protein expression levels. Human 1726 decreased the expression of IFNGR1 by approximately 71% and approximately 77% in HeLa and SH-SH57 cells, respectively (Figures S2A and S2B). Mouse 1641 decreased IFNGR1 by approximately 67% in N2a cells (Figure S2C).

The activation of IFN-γ signaling induces the expression of CXCL9/10/11.^31^ We further validated the lead compounds 1726 and 1641 in a cell model of IFN-γ signaling. Human HeLa and mouse N2a cells were treated with siRNA for 72 h followed by the stimulation of IFN-γ signaling using recombinant human or mouse IFN-γ. The mRNA levels of human *CXCL9/10/11* and mouse *Cxcl9/10/11* were measured 6 h after the stimulation. We observed a strong mRNA upregulation of human *CXCL9/10/11* (approximately 20-, 580-, and 150-fold) and mouse *Cxcl9/10/11* (approximately 3-, 20-, and 3-fold) in IFN-γ-stimulated cells compared with unstimulated cells (Figures 1G and 1H). The percentage of mRNA silencing after siRNA treatment was determined. Human 1726 significantly decreased the expression of *CXCL9* (approximately 77%; p < 0.05), *CXCL10* (approximately 64%; p < 0.05), and *CXCL11* (approximately 48%; p < 0.01) compared with untreated cells, whereas the non-targeting control (NTC) compound was not effective. Similarly, mouse 1641 significantly reduced the expression of *Cxcl9* (approximately 85%; p < 0.05), *Cxcl10* (approximately 90%; p < 0.001), and *Cxcl11* (approximately 82%; p < 0.01). These results indicate lead compounds 1726 and 1641 can efficiently inhibit IFN-γ signaling *in vitro*. Additionally, we compared mouse siRNA ^*Ifngr1*^ 1641 with ruxolitinib,^32^ an US Food and Drug Administration (FDA)-approved small molecule JAK1/2 inhibitor, for inhibiting IFN-γ signaling in the cell model. siRNA ^*Ifngr1*^ 1641 showed comparable potency as ruxolitinib in inhibiting *Cxcl9/10/11* mRNA expression (Figure S3). Mouse siRNA ^*Ifngr1*^ 1641 was used for subsequent *in vivo* studies.

### DCA conjugation enables efficient silencing of *Ifngr1* in skin local to the injection site

**Figure 2:**
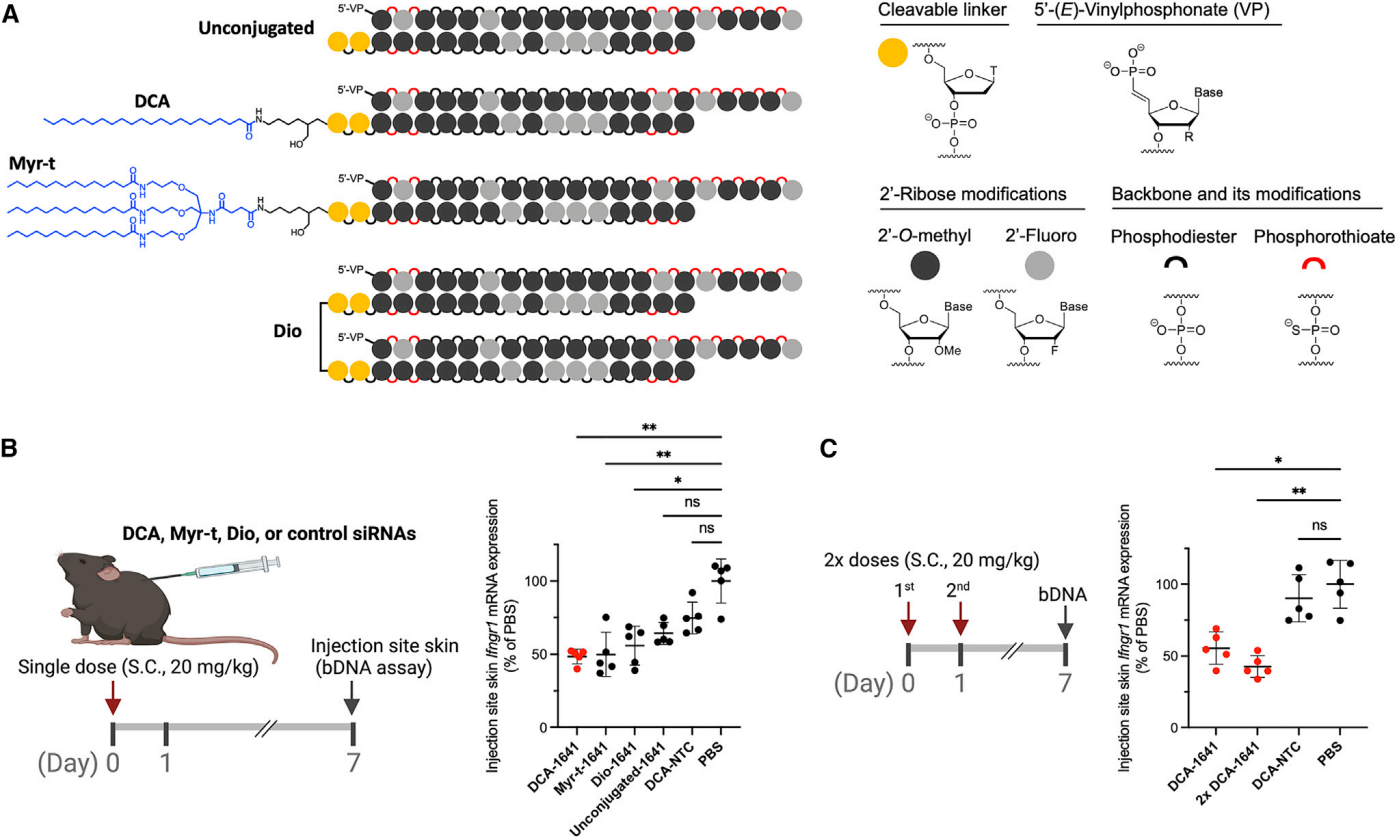
Chemical configurations of siRNA ^*Ifngr1*^ 1641 for gene silencing in mouse skin (A) Schematic of the chemical structures of hydrophobically conjugated (DCA; tri-myristic acid, Myr-t) and Dio siRNAs; DCA and Myr-t conjugates are covalently linked to the 3′ end of sense strand; the two sense strands of the Dio scaffold are covalently linked by a tetraethylene glycol; the study also included unconjugated siRNA ^*Ifngr1*^ 1641 and DCA conjugated NTC siRNAs. (B) *Ifngr1* mRNA silencing in skin at the injection site; mice (n = 5 per group) were injected subcutaneously (between shoulders) with a single dose of siRNA (20 mg/kg); local skin was collected at 1 week after injection and mRNA levels were measured using QuantiGene 2.0 assays; *Ifngr1* expression was normalized to a housekeeping gene *Ppib*; data are represented as percent of PBS control (mean ± standard deviation) and analyzed by Kruskal-Wallis test (∗p < 0.05, ∗∗p < 0.01; ns, not significant). (C) Efficacy of a single dose versus two doses (2×, 24 h apart; n = 5) of DCA-siRNA ^*Ifngr1*^ 1641 (One-way ANOVA, ∗p < 0.05, ∗∗p < 0.01). bDNA, branched DNA.

The chemical nature of the siRNA conjugate greatly impacts the tissue retention and efficacy of siRNAs.^23^^,^^24^^,^^33^ In our previous work, we evaluated a panel of lipid-conjugated compounds and found that highly hydrophobic conjugates enhance the local retention of siRNAs in skin at the injection site, whereas unconjugated compounds are cleared quickly. Among the hydrophobic conjugates, DCA, and tri-myristic acid (Myr-t) exhibited distinct therapeutic profile of safety, local retention, and silencing efficacy in skin.^23^ Therefore, to achieve local skin delivery of siRNA ^*Ifngr1*^ 1641 in this study, we covalently attached DCA or Myr-t to the 3′ end of the sense strand. We also included a divalent (Dio) siRNA scaffold to the evaluation panel (Figure 2A), as we have found that compound size may affect local retention and efficacy, for example, in the central nervous system.^34^

To compare the efficacy of the three chemical configurations, we subcutaneously injected wild-type C57BL/6J mice (n = 5) with a single dose of siRNA (20 mg/kg). The *Ifngr1* mRNA levels in skin local to the injection site were quantified 1 week after injection. As expected, all three configurations significantly reduced the *Ifngr1* expression by approximately 40%–50% (p < 0.05 or 0.01) compared with PBS (Figure 2B). Among them, DCA supported a slightly better efficacy than Myr-t and Dio. In comparison, NTC siRNA was not active and unconjugated siRNA ^*Ifngr1*^ 1641 induced minimal silencing that did not reach statistical significance (p > 0.05) (Figure 2B). Based on these data, we chose DCA-siRNA ^*Ifngr1*^ 1641 for subsequent studies.

We next evaluated whether two doses of DCA-siRNA ^*Ifngr1*^ 1641 could improve local *Ifngr1* silencing. Mice were injected with a single dose or two doses of compounds over 2 days (24 h apart), and the *Ifngr1* mRNA levels were measured 1 week after injection. The additional dose produced slightly better silencing (approximately 57% [p < 0.01] compared with PBS), but was not statistically different from the single dose (approximately 45% silencing) (Figure 2C). This result suggests improvement in local silencing efficacy is insignificant with multiple injections over a short period, consistent with our previous work.^25^ DCA-siRNA also supports systemic delivery to several tissues and the silencing in each tissue is target dependent.^23^ In this experiment, we simultaneously checked the systemic silencing of *Ifngr1* in liver, kidney, spleen, muscle, and skin (distal to injection site). DCA-siRNA ^*Ifngr1*^ 1641 exhibited a significant systemic effect on decreasing *Ifngr1* mRNA in liver (approximately 40–50%), but not in other tested organs (Figure S4).

### DCA-siRNA accumulates in major cell types of epidermis and dermis

**Figure 3:**
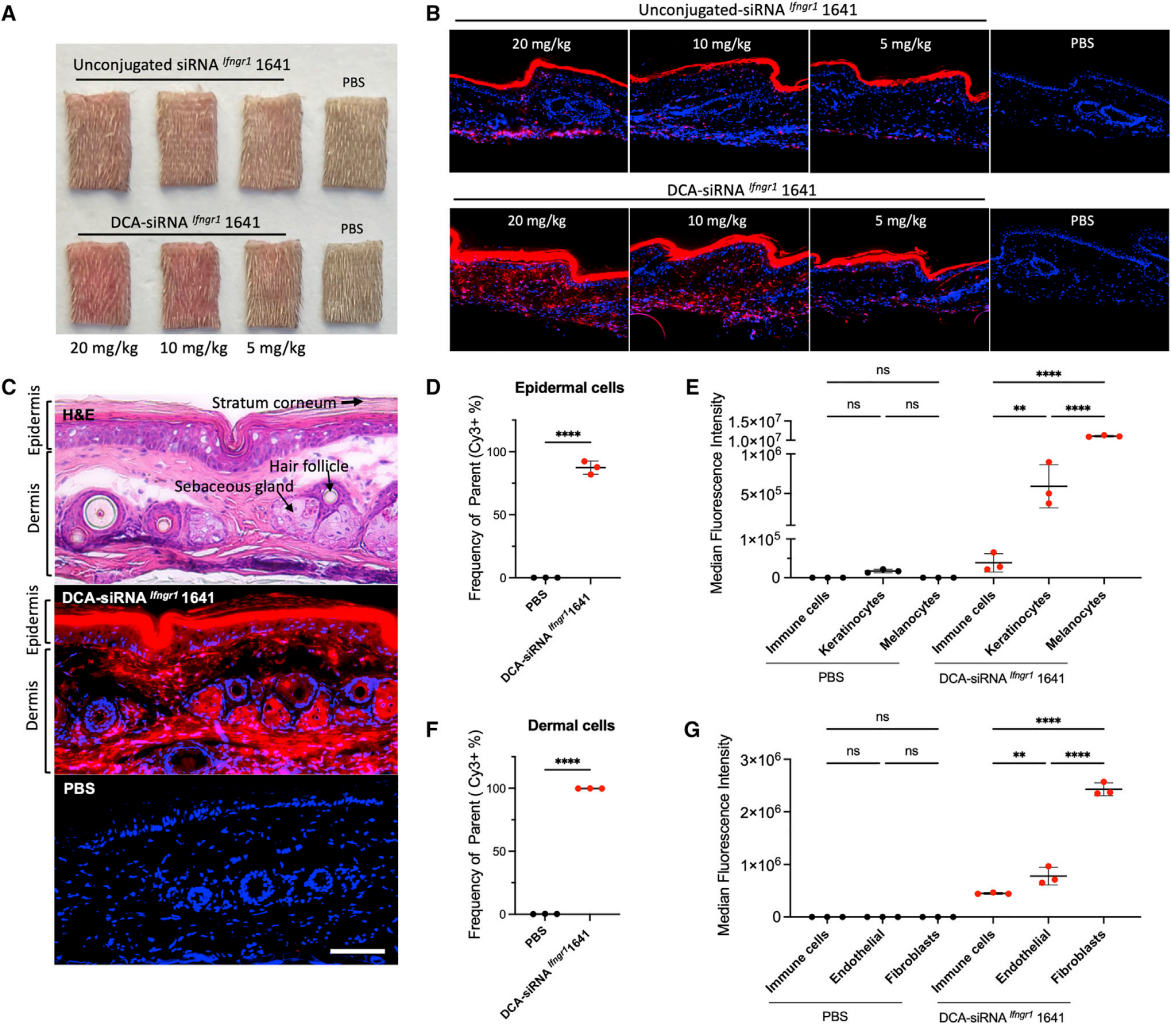
Local distribution profile of DCA-siRNA in skin cell types Single injection of Cy3-labeled siRNA ^*Ifngr1*^ 1641 with or without DCA conjugation (at 3 different doses: 20, 10, 5 mg/kg in 150 μL PBS) were subcutaneously injected into mouse tail skin (a 25G, 40-mm needle was fully inserted and pulled slowly while injecting to cover approximately two-thirds of the area of the tail). Skin samples were collected at 48 h after the injection. (A) Images of skin biopsies from the injection site show local retention of siRNAs (pink), and (B) fluorescence microscopic images show siRNA (red) retention in local skin (nuclei were stained in blue with DAPI, original magnification ×20). (C) Hematoxylin and eosin staining (top) of skin biopsy distinguishes the morphology of epidermis and dermis (transverse section, arrows indicate stratum corneum, sebaceous gland, and hair follicle); Cy3-DCA-siRNA ^*Ifngr1*^ 1641 distribution (red in middle panel); PBS background staining (bottom panel); original magnification ×20, bar scale = 100 μm. (D) Percentage of Cy3-positive cell population in live epidermal cells (n = 3, mean ± standard deviation, 20 mg/kg DCA-siRNA ^*Ifngr1*^ 1641, and PBS background control, unpaired t-test, ∗∗∗∗p < 0.0001), and (E) cell size-normalized median fluorescence intensity (MFI) indicates relative delivery efficiency of Cy3-siRNA in epidermal cell types (one-way ANOVA for multiple comparison; ∗p < 0.05, ∗∗∗∗p < 0.0001; ns, not significant). (F) Percentage of Cy3 positive cell population in live dermal cells, and (G) normalized MFI in dermal cell types (∗∗∗p < 0.001, ∗∗∗∗p < 0.0001).

To determine the delivery efficiency of DCA-siRNA in skin, we subcutaneously injected Cy3-labeled unconjugated or DCA conjugated siRNA ^*Ifngr1*^ 1641 into the tail skin of mice. Using tail skin enabled efficient sample processing for imaging and flow cytometry analysis. Skin biopsies from the local injection site were collected 48 h after injection. We observed an improved local retention of DCA-siRNAs (pink with Cy3 labeling) compared with unconjugated compounds (Figure 3A). The 20× fluorescence microscopic images also showed DCA-siRNAs were better retained at the skin of the injection site compared with unconjugated compounds (Figure 3B). This result supports the observation that DCA-siRNA ^*Ifngr1*^ 1641 was more effective in silencing *Ifngr1* mRNA than unconjugated compound locally (Figure 2B).

The efficiency of cellular uptake of chemically modified siRNAs varies by tissue and cell types.^35^ DCA-siRNA ^*Ifngr1*^ 1641 showed a broad local distribution into skin cells of the epidermis and dermis (Figure 3C). To analyze the cell type-specific accumulation of DCA-siRNA in skin, we stained the epidermal hematopoietic cells, melanocytes, and keratinocytes, as well as dermal hematopoietic cells, fibroblasts, and endothelial cells using a panel of molecular markers (CD45, CD49f, CD140a, and Ckit), then performed flow cytometry. Detailed gating and analysis strategies are shown in Figures S5 and S6. In general, DCA-siRNA transduced approximately 87% of epidermal cells and 100% of dermal cells (Figures 3D, 3F, S5B, and S6B). In epidermis, more than 95% hematopoietic cells, approximately 85% of keratinocytes, and 100% melanocytes were Cy3-siRNA positive (Figure S5B). The small but measurable fraction of hematopoietic cells (approximately 5%) and keratinocytes (approximately 15%) showing limited siRNA cellular uptake might be caused by the cornification process of superficial keratinocytes that limits the permeability and membrane fluidity of these cells for siRNA delivery. In contrast, all major dermal cell types were Cy3-siRNA positive (Figure S6B), indicating full accessibility of siRNA delivery in dermis. We also compared the relative delivery efficiency of siRNA with each cell type based on their population fluorescence intensity (Figures 3E and 3G). In epidermis, cell type delivery efficiency follows the order of melanocytes > keratinocytes > hematopoietic cells in Cy3-siRNA positive populations. In the dermis, the order is fibroblasts > endothelial > hematopoietic cells.

### A single dose of DCA-siRNA ^*Ifngr1*^ 1641 supports efficient IFNGR1 protein reduction in the skin for at least 4 weeks

**Figure 4:**
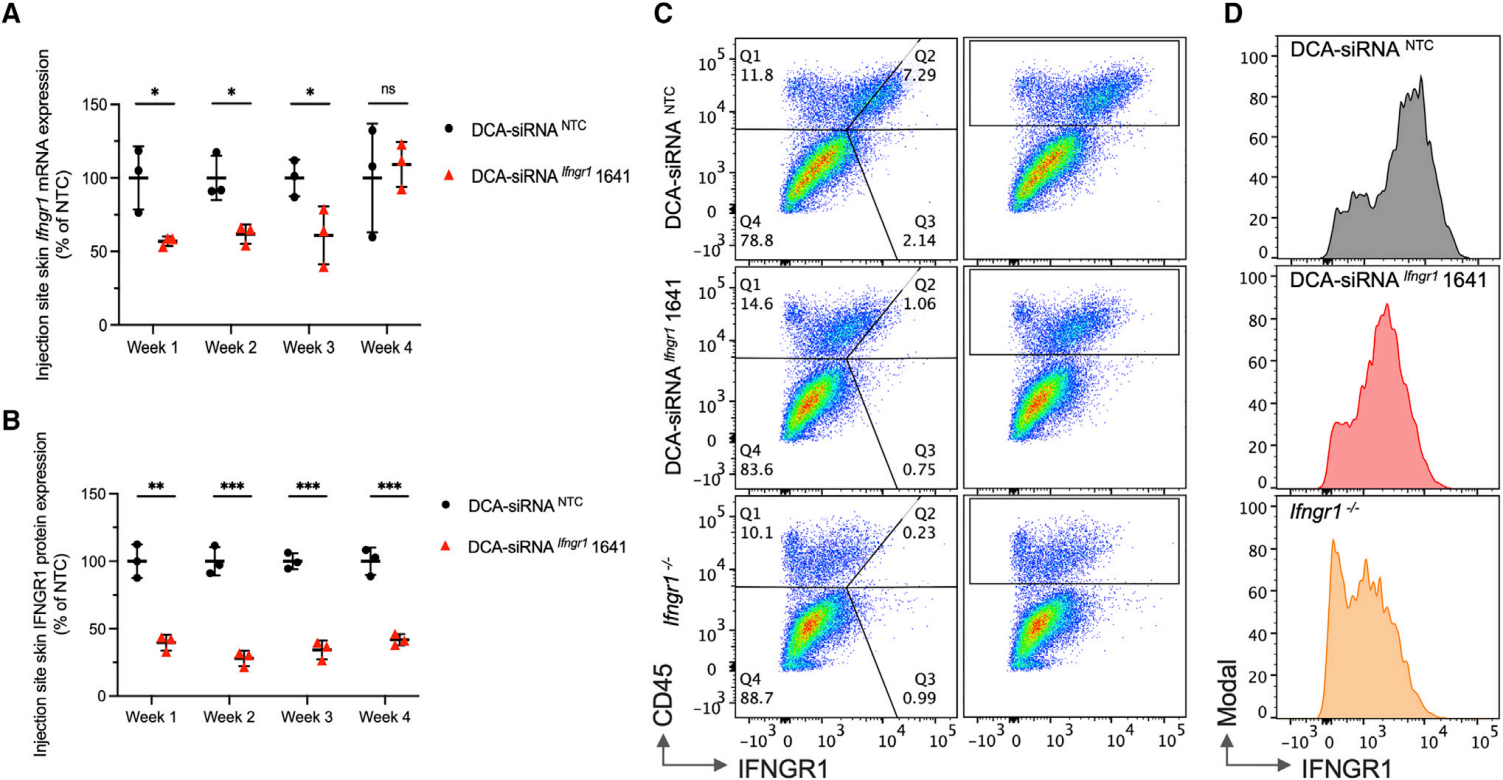
Duration of *Ifngr1* silencing by siRNA ^*Ifngr1*^ 1641 in mouse tail skin (A) *Ifngr1* mRNA levels over four weeks post injection (n = 3 per group, single dose at 20 mg/kg); mRNA levels were measured using QuantiGene 2.0 assays, *Ifngr1* expression was normalized to the housekeeping gene *Ppib*; data were represented as percent of NTC control (mean ± standard deviation) and analyzed by unpaired t test (∗p < 0.05; ns, not significant). (B) IFNGR1 protein levels were determined by the relative MFI of anti-mouse IFNGR1 staining in CD45^+^ (hematopoietic cell marker) cell population; MFI values were subtracted from Ifngr1^−/−^ background staining, and normalized to NTC control (mean ± standard deviation, unpaired t test, ∗∗p < 0.01, ∗∗∗p < 0.001). (C) Representative flow cytometry panels of CD45^+^ cells and IFNGR1 staining at week 2 post-injection. (D) Histograms of graph (C) for IFNGR1 staining in CD45^+^ cells.

To evaluate the duration of effect of DCA-siRNA ^*Ifngr1*^ 1641, we subcutaneously injected mice with a single dose (20 mg/kg) of compound in tail skin. Skin *Ifngr1* mRNA levels at the injection site were measured over 4 weeks. We observed a significant decrease in *Ifngr1* mRNA (approximately 40%–50%) in the skin of mice treated with DCA-siRNA ^*Ifngr1*^ 1641 from week 1 to week 3 compared with the NTC control (p < 0.05), but the mRNA silencing effect was lost at week 4 (Figure 4A). This shorter duration of effect may be due to active proliferation of skin cells. Thus, repeat injections with a 2- to 3-week interval might be necessary to maintain a maximum long-term *Ifngr1* silencing in skin.

The effect of DCA-siRNA ^*Ifngr1*^ 1641 on IFNGR1 protein decrease in skin cells was determined by fluorescence flow cytometry. Membrane expression of IFNGR1 can be found in multiple cell types and is relatively high in a subset of hematopoietic cells, such as in macrophage and dendritic cells.^36^, ^37^, ^38^ Flow cytometry revealed that IFNGR1 is mainly expressed in CD45^+^ hematopoietic cells under normal physiological conditions, with a higher staining intensity in dermis compared with epidermis (Figure S7). To provide a more reliable platform for data analysis, we determined the median fluorescence intensity of IFNGR1 in dermal CD45^+^ hematopoietic cells in the DCA-siRNA ^*Ifngr1*^ 1641-treated group relative to the NTC-treated group (Figures 4B–4D). Surprisingly, the silencing effect on protein level seems to be rapid (at week 1) and relatively stable, with a maximal decrease (approximately 70%) of IFNGR1 protein occurring at week 2. Notably, even at week 4, DCA-siRNA ^*Ifngr1*^ 1641-treated skin showed a sustained IFNGR1 decrease (approximately 60%) (Figure 4B).

### DCA-siRNA ^*Ifngr1*^ 1641 efficiently decreases the effects of IFN-γ signaling

**Figure 5:**
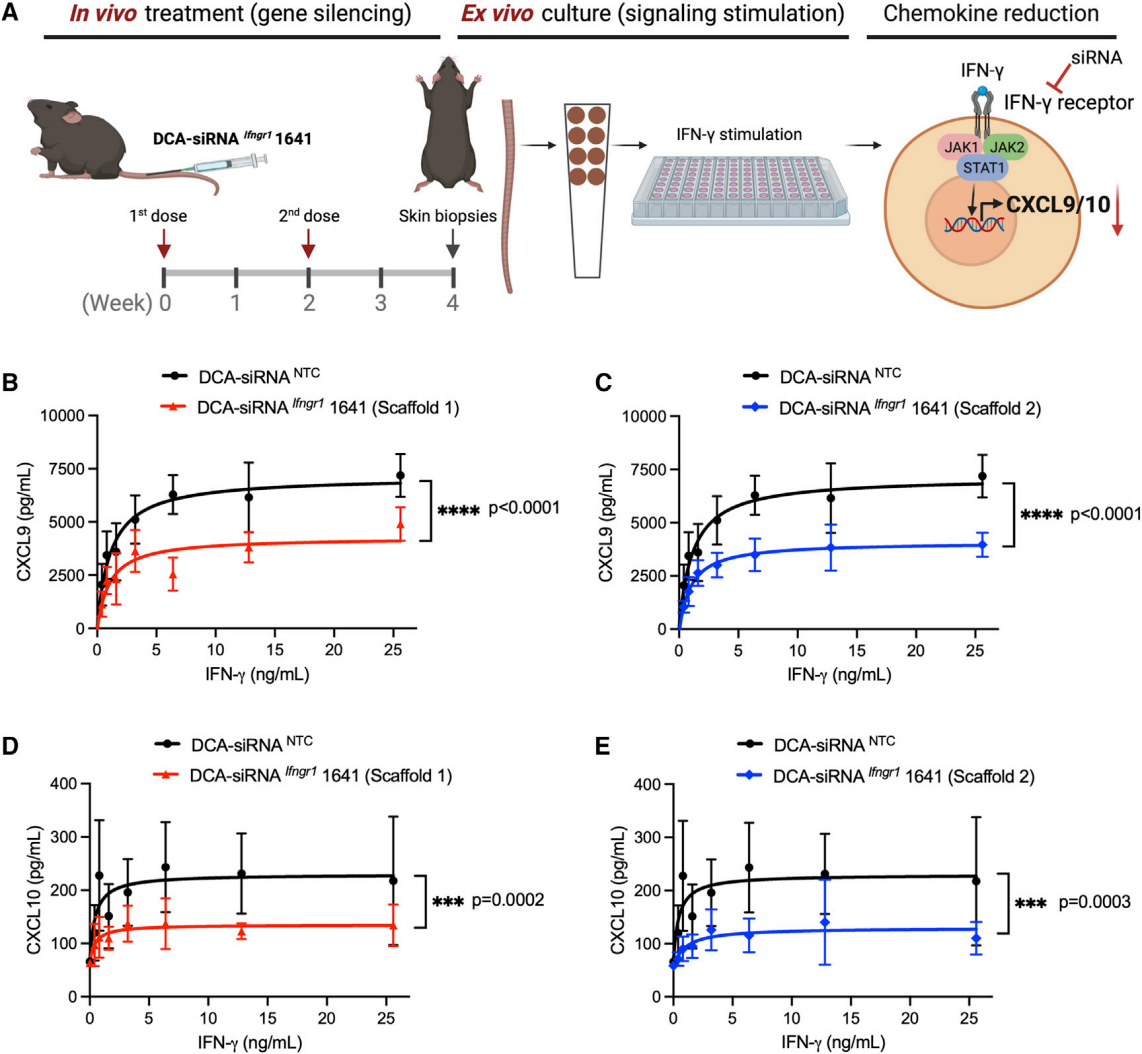
DCA-siRNA ^*Ifngr1*^ 1641 inhibits IFN-γ signaling in an *ex vivo* skin model (A) Schematic of siRNA treatment in mice and stimulation of IFN-γ signaling in an *ex vivo* skin biopsy model; mice (n = 5 per group) were treated subcutaneously with two doses (2×; 20 mg/kg; 2 weeks apart) of DCA-siRNA ^*Ifngr1*^ 1641 in tail skin, and eight punches per mouse of skin biopsies (4 mm in diameter) were collected. For each mouse, a seven-point dose response of IFN-γ signaling stimulation was carried out using recombinant mouse IFN-γ at a concentration range of 0–25.6 ng/mL. Skin biopsies were incubated at 37°C for 24 h, and CXCL9 and CXCL10 levels were determined by ELISA. (B–E) Production of CXCL9 and CXCL10 after the treatment of two scaffold configurations of DCA-siRNA ^*Ifngr1*^ 1641 (two-way ANOVA, ∗∗∗p < 0.001, ∗∗∗∗p < 0.0001).

To determine whether the silencing of *Ifngr1* by DCA-siRNA ^*Ifngr1*^ 1641 translates to a functional inhibition of IFN-γ signaling in skin, we developed an *ex vivo* skin culture model that enables direct measurement of IFN-γ-driven chemokine levels in culture media by using ELISAs (Figure 5A). In this model, mice were subcutaneously injected with two doses (20 mg/kg, 2 weeks apart) of DCA-siRNA ^*Ifngr1*^ 1641 in tail skin, and skin biopsies were taken from the injection site 2 weeks after the second dose. We then incubated the skin biopsies with mouse recombinant IFN-γ to induce CXCL9 and CXCL10 expression. Mature CXCL11 is not produced in wild-type C57BL/6J mice owing to a frameshift mutation of the *Cxcl11* gene.^39^, ^40^, ^41^ A critical step in optimizing siRNAs for a specific siRNA sequence is to test varying patterns of chemically modified nucleotides, including 2′-OMe, 2′-F, and backbone PS linkages. In this skin culture model, we tested the efficacy of an additional siRNA scaffold, defined as scaffold 2, along with the scaffold 1, which was used in the previous experiments outlined in this article. The detailed information of modification patterns is shown in Table S4. compared with NTC control, treatment with scaffold 1 significantly decreased CXCL9 expression by approximately 32% (Figure 5B) (p < 0.0001), whereas scaffold 2 decreased CXCL9 levels by approximately 45% (Figure 5C) (p < 0.0001). Comparably, scaffold 1 significantly reduced CXCL10 expression by approximately 38% (Figure 5D) (p = 0.0002), whereas scaffold 2 decreased CXCL10 levels by approximately 49% (Figure 5E) (p = 0.0003). This is consistent with our additional studies (data not shown) showing that scaffold 2 is slightly more potent than scaffold 1 in the context of certain siRNA sequences. Taken together, these data demonstrate that local administration of DCA-siRNA ^*Ifngr1*^ 1641 leads to efficient IFN-γ signaling inhibition in skin.

## Discussion

Rapid discovery and optimization of drug candidates remain challenging. The success of a therapeutic modality is defined by (1) its ability to affect the target of interest and (2) its pharmacokinetic behavior. For small molecules, these characteristics are inseparable, necessitating a unique, iterative optimization process for each drug candidate. Antibodies typically only efficiently bind to the cell surface or secreted proteins. By contrast, the development of therapeutic siRNAs is not limited by these challenges and is highly programmable—the sequence defines the specificity, while the siRNA scaffold and chemical modifications dictate pharmacokinetics.^17^ With recent advances in oligonucleotide chemistry and *in vivo* delivery, the FDA has approved the use of several siRNA drugs (e.g., patisiran for hATTR amyloidosis-PN, givosiran for acute hepatic porphyria, lumasiran for primary hyperoxaluria type 1, and inclisiran for hypercholesterolemia),^18^, ^19^, ^20^^,^^42^ with additional candidates currently in late-stage clinical trials. The adaptability of RNAi-based drugs has the potential to transform future therapeutic development.

Here, we identify siRNAs that target immunomodulatory pathways, outlining an efficient approach for developing siRNA therapeutics for the local modulation of gene expression in skin. Dysregulation of IFN-γ signaling is associated with many inflammatory and autoimmune diseases,^37^^,^^38^^,^^43^ and modulation of IFN-γ signaling pathway using small molecule JAK inhibitors show encouraging results for treating some skin diseases.^9^, ^10^, [11], 12, 13 We hypothesize that targeting IFN-γ receptor may prove to be a viable and more specific strategy for modulating IFN-γ signaling. By combing RNAi bioinformatics, chemical biology, and *in vivo* pharmacology, this pilot study identifies siRNAs that support efficient and sustained reduction of IFNGR1 in skin. Similarly, the DCA-siRNA framework could be applied to other targets of interest in skin. As IFN-γ signaling is involved in many pathological processes, the functional siRNAs presented in this work could be further developed for a wide range of therapeutic applications in tissues beyond skin.

Identifying siRNA compounds that target both human and mouse mRNA transcripts is ideal, but sometimes gene homology differs significantly across species, providing limited opportunity for cross-reactive siRNA design. This is particularly true for genes involved in immunomodulation. Indeed, we tested several cross-reactive compounds that were active in mouse cells, but not in human cells. Generally, compound activity is similar in different cell types across species; however, this is not always the case.^44^ The subcellular environment, localization, secondary structure, and protein binding state of mRNA all affect the efficacy of siRNAs. For example, a previous study reported that siRNAs are more effective for silencing mRNAs are under active translation; this is because ribosomes can alleviate the masking effect of mRNA secondary structures to improve siRNA accessibility.^45^ One explanation for the limited efficacy of the cross-reactive siRNAs in human cells is that human *IFNGR1* mRNAs may contain more higher-order structures compared with their mouse counterparts. When the discovery of cross-reactive candidates is unsuccessful, an alternative strategy is to identify one set of compounds with homology to human and non-human primates, and another set of tool compounds that can be used in rodent studies, as we have done here.

We found that DCA and Myr-t conjugates as well as the Dio scaffold support the efficacy of siRNA ^*Ifngr1*^ at the injection site skin, whereas unconjugated compounds provide only minimal efficacy. Lipid conjugation greatly increases hydrophobicity of siRNA duplexes and, thus, enhances the local retention of injected siRNAs.^23^^,^^24^ Also, covalently combining two siRNAs to form a Dio scaffold might significantly decrease the clearance rate of the compound owing to the larger size of the molecule. Thus, fine-tuning the conjugate chemistry and scaffold valency may represent an efficient strategy to manipulate the retention and potency of siRNAs in skin for therapeutic applications.

Our skin biodistribution data reveal that DCA-conjugated siRNAs are more (multiple-fold higher) efficiently distributed into epidermal melanocytes and dermal fibroblasts than into other cell types. Although we have not investigated the selective silencing of genes that are preferentially expressed in melanocytes and fibroblasts, the distribution profile could be used for the functional delivery of DCA-siRNAs to these cells while limiting siRNA exposure to other cell types. One possible strategy is to determine a lower dose that maintains the efficacy of DCA-siRNAs in melanocytes and fibroblasts while decreasing siRNA effects in other cell types for improving the cell-type specificity of the compounds.

We observed a difference in the kinetics of *in vivo* silencing between IFNGR1 mRNAs and proteins. Maximal mRNA silencing in the skin was achieved at week 1, decreased by week 3, and returned to control levels by week 4. At the protein level, the degree of silencing peaked at week 2 and was maintained for at least 4 weeks. The observed difference in kinetics is likely due to the relatively slow turnover rate of IFNGR1 protein in skin, resulting in a longer efficacy from the siRNA treatment.

Human *IFNGR1* gene deficiency has been associated with an increased susceptibility to certain infectious diseases in patients, such as mycobacterial infections.^46^, ^47^, ^48^ This raises questions about the systemic effects of DCA-siRNA from silencing *IFNGR1*. In our work, DCA-siRNA ^*Ifngr1*^ show systemic silencing of mouse *Ifngr1* in the liver, but not in the kidneys, spleen, muscle, or distal skin. A downregulation of IFN-γ signaling in the liver may be beneficial or deleterious depending on the pathophysiological conditions of liver as IFN-γ is a cytokine known for its broad immunomodulatory properties, such as inducing hepatocyte apoptosis, inhibiting hepatocyte cell cycle progression, and activating antiviral responses as previously discussed in detail by Horras et al.^49^ Thus, safety precautions should be considered in targeting IFN-γ signaling pathway in the long term. Additionally, DCA-siRNA ^*Ifngr1*^ might exhibit systemic effects besides the liver, as it has not been fully characterized owing to the limited scope of this work. We previously reported the tissue accumulation levels of DCA-siRNAs,^23^ but the pharmacokinetics have not been established. A comprehensive study focused on the tissue expression profile of *IFNGR1*, pharmacokinetic properties of DCA-siRNA ^*Ifngr1*^, and levels of target silencing would provide insight into the safety and therapeutic potentials of the DCA-siRNA ^*Ifngr1*^ in extracutaneous tissues.

We have demonstrated that *IFNGR1* siRNAs efficiently decrease the production of IFN-γ-inducible chemokines CXCL9/10/11 upon IFN-γ signaling activation. The CXCL9/10/11-CXCR3 axis regulates immune cell migration, differentiation, and activation, leading to various immunoregulatory effects in many diseases, such as autoimmune disorders, infections, and cancers.^5^^,^^7^^,^^31^^,^^43^^,^^50^ Therefore, modulating IFN-γ signaling may positively or negatively impact the biological functions of CXCL9/10/11-CXCR3 axis during the progression of these diseases. A better understanding of the advantages and limitations of silencing IFN-γ signaling under a defined disease state would provide a clearer clinical relevance of our strategy using the identified *IFNGR1* siRNAs.

Future studies should also explore how the routes of administration (e.g., controlled epidermal penetration, intradermal, or subcutaneous) and dosing regimens can be optimized to achieve selective gene silencing in different skin layers, especially in a skin model that closely resembles human skin morphology and functionality, such as in porcine skin. The functional delivery of siRNAs remains one of the main challenges of modulating a gene of interest in skin. Current methods under development to overcome the barriers of skin delivery of macromolecules include microneedle patches, skin-penetrating microparticles, ionic-liquid formulations, and others.^51^, ^52^, ^53^, ^54^ The delivery of validated siRNAs into skin with patient-friendly strategies would greatly potentiate the clinical implementation of RNAi-based therapeutics in skin diseases. Taken together, our work demonstrates the rational design of a targeted siRNA therapy and establishes a path toward a new treatment paradigm for skin diseases.

## Materials and methods

### Oligonucleotide synthesis

Modified siRNAs were synthesized using standard solid phase phosphoramidite chemistry on a Dr. Oligo 48 medium throughput (Biolytic) or MerMade 12 (BioAutomation) oligonucleotide synthesizer. We used 2′-O-methyl, 2′-fluoro, and custom 5’-(E)-vinylphosphonate phosphoramidites (Chemgenes) for strand stabilization. The sense strands of compounds for *in vitro* screens were synthesized at 1-μM scale on a cholesterol-conjugated solid support (Chemgenes), and the sense strands of compounds for *in vivo* injection were synthesized at 10-μM scale on a DCA-functionalized controlled pore glass (CPG) as previously described.^23^, ^24^, ^25^ Cy3 phosphoramidites (Gene Pharma) were used for fluorescence labeling of the 5′ of sense strands. Antisense strands were synthesized on CPG functionalized with a Unylinker (Chemgenes), bis-cyanoethyl-N, N-diisopropyl CED phosphoramidite (Chemgenes) was used to introduce a 5′-monophosphate on antisense strands for *in vitro* experiments, and 5’-(E)-vinylphosphonate modification was applied to antisense strands for *in vivo* studies. Sense strands were cleaved and deprotected using 40% aq. methylamine and 30% NH_4_OH (1:1) at room temperature for 2 h, and antisense strands were deprotected with a solution of bromotrimethylsilane:pyridine (3:2, v/v) in dichloromethane (5 mL) for the (E)-vinylphosphonate deprotection, then cleaved and deprotected with 30% NH_4_OH containing 3% diethylamine at 35°C for 20 h. The deprotected oligonucleotide solutions were filtered to remove CPG residues. The filtrates were frozen in liquid nitrogen shortly and dried by a SpeedVac vacuum centrifuge. The resulting oligonucleotide pellets were reconstituted in 5% acetonitrile in water for subsequent purifications.

### HPLC purification

The purification of oligonucleotides was performed on an Agilent 1290 Infinity II system. Sense strands were purified on a Hamilton PRP-C18 reverse phase column using the following conditions: eluent A, 50 mM sodium acetate in 5% acetonitrile; eluent B, 100% acetonitrile; gradient 0%–20% for 3 min, 20%–70% for 23 min, followed by cleaning and recalibration for 9 min; column temperature, 60°C; and flow rate, 40 mL/min. Antisense strands were purified over anion-exchange column (GE Source 15Q media) using the following conditions: eluent A, 10 mM sodium acetate in 20% acetonitrile; eluent B, 1 M sodium perchlorate in 20% acetonitrile; gradient 0%–20% for 3 min, 20%–70% for 23 min, followed by cleaning and recalibration for 9 min; column temperature, 60°C; and flowrate, 40 mL/min. Peaks were monitored by UV absorbance at 260 nm. The pure oligonucleotide fractions were collected and characterized by liquid chromatography mass/mass spectrometry (LC/MS). The fractions were combined, frozen, and dried in a Speed Vac overnight. Purified oligonucleotides were desalted by size-exclusion chromatography (GE Sephadex G-25 fine), and lyophilized.

### LC/MS analysis

The purity and identity of all oligonucleotides used in the studies were characterized on an Agilent 6530 accurate mass Q-TOF LC-MS using reverse phase chromatography under the following conditions: mobile phase A: 9 mM triethylamine/100 mM hexafluoroisopropanol in water; mobile phase B: 9 mM triethylamine/100 mM hexafluoroisopropanol in methanol; temperature, 60°C; flow rate, 0.5 mL/min UV, 260 nm; LC column, Agilent 2.1 × 50 mm AdvanceBio C18 oligonucleotide column. MS parameters: source, electrospray ionization; ion polarity, negative mode; range, 100–3,200 m/z; scan rate, 2 spectra s^−1^; capillary voltage, 4,000; fragmentor, 180 V.

### Cell culture and *in vitro* screen

HeLa cells (ATCC; #CCL-2) were maintained in DMEM (Corning Cellgro; #10–013CV), and Neuro-2a (N2a) cells (ATCC; #CLL-131) were maintained in EMEM (ATCC; #30–2003). The media were supplemented with 10% fetal bovine serum (FBS, Gibco; #26140), and all cells were grown at 37°C and 5% CO_2_. *In vitro* screen was carried out as previously described.^44^^,^^55^ Briefly, HeLa and N2a cells were treated with cholesterol-conjugated siRNA for 72 h in 50/50 (vol/vol) of 6% FBS medium/Opti-MEM (Gibco, #31985–079) without antibiotics. Cells were then lysed in diluted lysis mixture consisting of a 1:2 ratio of lysis mixture (Invitrogen, #13228): water with 0.2 mg/mL of proteinase K (Invitrogen, #25530–049) at 55°C for 30 min mRNA expression was measured by the Quantigene 2.0 assay (Affymetrix) with the following probe sets (ThermoFisher Scientific): human *IFNGR1* (#SA-3001758), human *PPIB* (#SA-10003) or human *HPRT* (#SA-10030); mouse *Ifngr1*(#SB-3029581), mouse *Ppib* (#SA-10002) or mouse *Hprt* (#SA-15463).

### *In vitro* model of IFN-γ signaling

Human HeLa cells and mouse N2a cells were treated with 1.5 μM of siRNA at 37°C for 72 h as in screen experiments. After 72 h, medium was replaced with new medium containing 10 ng/mL of recombinant human IFN-γ protein (R&D System, #285-IF-100) and 10 ng/mL recombinant human tumor necrosis factor (TNF)-α protein (R&D System, #210-TA-020) to stimulate IFN-γ signaling in HeLa cells. For stimulating IFN-γ signaling in N2a cells, 10 ng/mL of recombinant mouse IFN-γ protein (R&D System, #485-MI-100) and 10 ng/mL recombinant mouse TNF-α protein (R&D System, #410-TRNC-010) were used. After 6 h of signaling stimulation, cells were lysed as in screen experiments. Expression of mRNA was measured by the Quantigene 2.0 assay with the following probesets (ThermoFisher Scientific): human *CXCL9* (#SA-12372), human *CXCL10* (#SA-50393), human *CXCL11* (#SA-50464), human *ACTB* (#SA-10008); mouse *Cxcl9* (#SB-3030147), mouse *Cxcl10* (#SB-10026), mouse *Cxcl11* (#SB-3033872), mouse *Actb* (#SB-10003). Ruxolitinib (JAK1 and 2 small molecule inhibitor, TOCRIS, #7064, MW 310.87, C_17_H_18_N_6_⋅1/4 H_2_O) was used for the inhibition of IFN-γ signaling in N2a cells.

### Mice

Animal studies were conducted in accordance with the guidelines of University of Massachusetts Chan Medical School Institutional Animal Care and Use Committee. All procedures were approved under the Protocol #202000010 (Khvorova Laboratory) and #201900330 (Harris Laboratory) and in accordance with the National Institutes of Health Guide for the Care and Use of Laboratory Animals. Wild-type C57BL/6J mice were purchased from The Jackson Laboratory, and were 8–10 weeks of age at the time of the experiments.

### *In vivo* mRNA silencing

Skins were collected at the indicated time points and stored in RNA later (Sigma-Aldrich, #R0901) at 4°C overnight. The mRNA level was quantified using QuantiGene Singleplex assay kit (Invitrogen, #QS0016). Five punches of 4-mm diameter biopsies were mechanically homogenized in 500 μL of homogenization buffer containing 0.2 mg/mL Proteinase K (Invitrogen, #AM3546), followed by an incubation at 55°C for 30 min. The skin homogenate was then centrifuged at 14,000×*g* for 5 min, and the supernatant was collected for subsequent assays. Diluted samples and probesets (mouse *Ifngr1*, mouse *Ppib*) were added to the branded DNA capture plate and signal was amplified as described previously.^30^ The luminescence signal was detected on a Tecan M1000 microplate reader.

### Fluorescence microscopy

Skin samples were freshly embedded into Optimal cutting temperature compound (Sakura Finetek) before frozen sectioning. Hematoxylin and eosin staining was used for histology. Nuclei were stained with DAPI. All fluorescent images were acquired with a Leica DMi8 inverted microscope (Leica Microsystems). Images were analyzed using the Leica LAS X.

### Flow cytometry

Mouse tail skins were harvested at the indicated time points and incubated with 2.5 mg/mL Dispase II (Roche, #04942078001) at 37°C for 1 h. Epidermis was isolated and mechanically dissociated. Dermis was further incubated with a cocktail solution containing 2.5 mg/mL of collagenase IV (Sigma-Aldrich, #C6885) and 1 mg/mL deoxyribonuclease I (Sigma-Aldrich, #DN25) in RPMI-1640 medium (Gibco, #11875093) at 37°C for 45 min, followed by mechanical dissociation. All samples were filtered with 100-μm filters before staining. For antibody staining, UltraComp eBeads (Invitrogen, #01-2222-42) and ArC amine reactive compensation bead kit (Invitrogen, #A10346) were used as compensation controls. Samples were blocked with Fc block 2.4 G2 (Bio X cells, # BE0307) and stained using Live/Dead Zombie Aqua (Biolegend, #423101) or Zombie NIR (Biolegend, #423105) fixable viability kits. The following antibodies were used at a 1:100 dilution: Biotin anti-mouse CD119 (Biolegend, #112803), APC streptavidin secondary Ab (Biolegend, #4052–7), FITC anti-mouse CD45 (TONBO biosciences, #35-0451-U500), Pacific Blue anti-mouse I-A/I-E (Biolegend, #107620), Brilliant Violet 570 anti-mouse CD45 (Biolegend, #103116), APC anti-mouse CD117 (c-Kit) (TONBO biosciences, #35-0451-U500), PerCP/Cy5.5 anti-human/mouse CD49f (Biolegend, #313618), and 1:10 dilution for FITC anti-mouse CD140a (Miltenyi Biotech, #130-109-735) as suggested. The data were collected under BD LSR II or CYTEK Aurora flow cytometer and analyzed by FlowJo 10.7 software.

### *Ex vivo* skin model of IFN-γ signaling

Wild-type C57BL/6J mice (n = 5) were injected subcutaneously with two doses of siRNA (20 mg/kg at weeks 0 and 2) in the tail skin. Four weeks later, eight biopsy punches per mouse (4 mm in diameter) from the injected area of skin were taken and stimulated with mouse recombinant IFN-γ (R&D System, #485-MI-100) at concentrations range from 0 to 25.6 ng/mL (two-fold serial dilutions) in a seven-point dose response treatment. The skin punches were cultured at 37°C for 24 h in DMEM: Nutrient Mixture F-12 (DMEM/F-12 in 3:1 ratio, 10% FBS) with the following components added: epidermal growth factor (10 ng/mL), insulin (5 μg/mL), adenine (24.3 μg/mL), cholera toxin (10 ng/mL), transferrin (5 μg/mL), and tri-iodo-L-thyronine (1.36 ng/mL). The culture media of skin biopsies were five-fold diluted for quantification of CXCL9 expression.

### ELISA

Cell studies: Human IFN-γ R1 ELISA kit (Invitrogen, #EH249RB) and mouse IFN-γ R1 ELISA kit (Invitrogen, #EM40RB) were used according to manufacturer’s instructions. Human HeLa, SH-SH5Y, and mouse N2a cells were seeded in a 24-well plate and treated with 500 μL siRNA at 1.5 μM for 72 h. Cells were lysed with 700 μL/well RIPA buffer with proteinase inhibitor cocktail (Roche, #11836153001). Total protein levels were quantified by Bradford assay kit (ThermoFisher Scientific, #23236).

Animal studies: Mouse CXCL9 DuoSet ELISA kit (R&D System, #DY492-05) and DuoSet Ancillary Reagent Kit 2 (R&D System, #DY008) were used according to manufacturer’s instructions. Optical densities were measured with a Tecan M1000 (Tecan) microplate reader, and the absorbance values at 540 nm were subtracted from 450 nm to calculate CXCL9 level using a standard curve derived from the known concentration of the recombinant proteins. Mouse CRG-2 (CXCL10) ELISA kit (Invitrogen, #EMCXCL10) was used according to manufacturer’s instructions. Optical densities at 450 nm were measured using a SpectraMax M5 microplate reader.

### Statistical analysis

Data were analyzed using GraphPad Prism 9 software (GraphPad Software, Inc). Detailed information for data analysis related to each figure can be found in figure legends.

## Supplemental information

Document S1. Figures S1–S7 and Tables S1–S4

Document S2. Article plus supplemental information
