# Supplementary material for: RNAi-based modulation of IFN-γ signaling in skin
Source: Mol Ther. 2022 Apr 27;30(8):2709–21. doi: 10.1016/j.ymthe.2022.04.019 (PMC9372319; doi:10.1016/j.ymthe.2022.04.019)
Supplement: Document S1. Figures S1–S7 and Tables S1–S4 [file mmc1.pdf]

YMTHE, Volume *30*

Supplemental Information

RNAi-based modulation of IFN-γ signaling in skin

Qi Tang, Jacquelyn Sousa, Dimas Echeverria, Xueli Fan, Ying-Chao Hsueh, Khashayar Afshari, Nicholas MeHugh, David A. Cooper, Lorenc Vangjeli, Kathryn Monopoli, Ken Okamura, Annabelle Biscans, Adam Clauss, John E. Harris, and Anastasia Khvorova

SUPPLEMENTAL INFORMATION

Table of Contents

Table S1. Human targeting siRNA sequences and modification patterns.

Table S2. Human and mouse cross-targeting siRNA sequences and modification patterns.

Table S3. Mouse targeting siRNA sequences and modification patterns.

Table S4. Sequences and modification patterns of the two DCA-siRNA ^{Ifngr1} 1641 scaffolds used for *in vivo* studies.

Figure S1. Dose response of top hits in silencing human *IFNGR1* and mouse *Ifngr1*.

Figure S2. IFNGR1 protein expressions in human HeLa, SH-SY5Y, and mouse N2a cells.

Figure S3. Comparison of siRNA ^{Ifngr1} 1641 with Ruxolitinib in inhibiting IFN-γ signaling.

Figure S4. Systemic silencing of *Ifngr1* in liver, kidney, spleen, distal muscle, and distal tail skin.

Figure S5. Gating strategy of cell populations in epidermis and Cy3-DCA-siRNA ^{Ifngr1} 1641 delivery in cell types.

Figure S6. Gating strategy of cell populations of dermis and Cy3-DCA-siRNA ^{Ifngr1} 1641 delivery in cell types.

Figure S7. IFNGR1 expression ratio is higher in $CD45^+$ hematopoietic cells of dermis than in epidermis.

Table S1. Human targeting siRNA sequences and modification patterns.

*IFNGR1*_NM_000416_375 [a]	P(mU)#(fG)#(mA)(mA)(mU)(fU)(mU)(mG)(mA)(mU)(mG)(mG)(mA)#(fU)#(mC)#(fA)#(mC)#(mC)#(mA)#(fA) [b]
	(mG)#(mA)#(mU)(mC)(fC)(fA)(fU)(mC)(fA)(mA)(mA)(mU)(mU)#(mC)#(mA)-TegChol [c]
*IFNGR1*_NM_000416_516	P(mU)#(fU)#(mC)(mA)(mA)(fU)(mC)(mA)(mU)(mG)(mA)(mU)(mU)#(fU)#(mG)#(fC)#(mU)#(mU)#(mC)#(fU)
	(mC)#(mA)#(mA)(mA)(fU)(fC)(fA)(mU)(fG)(mA)(mU)(mU)(mG)#(mA)#(mA)-TegChol
*IFNGR1*_NM_000416_821	P(mU)#(fA)#(mA)(mC)(mC)(fU)(mU)(mU)(mU)(mA)(mU)(mA)(mC)#(fU)#(mG)#(fC)#(mU)#(mA)#(mU)#(fU)
	(mC)#(mA)#(mG)(mU)(fA)(fU)(fA)(mA)(fA)(mA)(mG)(mG)(mU)#(mU)#(mA)-TegChol
*IFNGR1*_NM_000416_824	P(mU)#(fG)#(mA)(mG)(mA)(fA)(mC)(mC)(mU)(mU)(mU)(mU)(mA)#(fU)#(mA)#(fC)#(mU)#(mG)#(mC)#(fU)
	(mU)#(mA)#(mU)(mA)(fA)(fA)(fA)(mG)(fG)(mU)(mU)(mC)(mU)#(mC)#(mA)-TegChol
*IFNGR1*_NM_000416_1027	P(mU)#(fA)#(mA)(mA)(mU)(fG)(mG)(mC)(mU)(mG)(mG)(mU)(mA)#(fU)#(mG)#(fA)#(mC)#(mG)#(mU)#(fG)
	(mC)#(mA)#(mU)(mA)(fC)(fC)(fA)(mG)(fC)(mC)(mA)(mU)(mU)#(mU)#(mA)-TegChol
*IFNGR1*_NM_000416_1393	P(mU)#(fA)#(mC)(mC)(mU)(fU)(mU)(mA)(mU)(mU)(mA)(mU)(mU)#(fU)#(mG)#(fG)#(mG)#(mG)#(mG)#(fA)
	(mC)#(mA)#(mA)(mA)(fU)(fA)(fA)(mU)(fA)(mA)(mA)(mG)(mG)#(mU)#(mA)-TegChol
*IFNGR1*_NM_000416_1631	P(mU)#(fA)#(mU)(mA)(mA)(fU)(mC)(mU)(mU)(mU)(mU)(mC)(mA)#(fU)#(mG)#(fA)#(mA)#(mA)#(mU)#(fU)
	(mC)#(mA)#(mU)(mG)(fA)(fA)(fA)(mA)(fG)(mA)(mU)(mU)(mA)#(mU)#(mA)-TegChol
*IFNGR1*_NM_000416_1726	P(mU)#(fC)#(mC)(mA)(mA)(fA)(mA)(mG)(mU)(mG)(mA)(mA)(mA)#(fA)#(mU)#(fG)#(mC)#(mC)#(mA)#(fC)
	(mA)#(mU)#(mU)(mU)(fU)(fC)(fA)(mC)(fU)(mU)(mU)(mU)(mG)#(mG)#(mA)-TegChol
*IFNGR1*_NM_000416_1745	P(mU)#(fA)#(mA)(mA)(mG)(fU)(mC)(mU)(mG)(mU)(mA)(mC)(mU)#(fU)#(mU)#(fA)#(mC)#(mA)#(mA)#(fG)
	(mA)#(mA)#(mA)(mG)(fU)(fA)(fC)(mA)(fG)(mA)(mC)(mU)(mU)#(mU)#(mA)-TegChol
*IFNGR1*_NM_000416_1989	P(mU)#(fC)#(mC)(mA)(mA)(fU)(mU)(mU)(mU)(mG)(mA)(mA)(mA)#(fA)#(mG)#(fC)#(mU)#(mU)#(mG)#(fC)
	(mC)#(mU)#(mU)(mU)(fU)(fC)(fA)(mA)(fA)(mA)(mU)(mU)(mG)#(mG)#(mA)-TegChol
*IFNGR1*_NM_000416_2021	P(mU)#(fU)#(mU)(mA)(mA)(fU)(mU)(mC)(mU)(mA)(mU)(mU)(mA)#(fG)#(mU)#(fU)#(mU)#(mG)#(mA)#(fA)
	(mA)#(mC)#(mU)(mA)(fA)(fU)(fA)(mG)(fA)(mA)(mU)(mU)(mA)#(mA)#(mA)-TegChol
*IFNGR1*_NM_000416_2072	P(mU)#(fC)#(mU)(mA)(mA)(fC)(mU)(mG)(mU)(mA)(mA)(mU)(mG)#(fU)#(mU)#(fU)#(mC)#(mA)#(mU)#(fA)
	(mA)#(mA)#(mC)(mA)(fU)(fU)(fA)(mC)(fA)(mG)(mU)(mU)(mA)#(mG)#(mA)-TegChol

[a] Target name_accession number_mRNA binding site; Database: (NCBI: NM_000416)

[b] siRNA antisense strand sequence and modifications. m=2’-O-methyl; f=2’-Fluoro; #=Phosphorothioate; P=5’-Phosphate

[c] siRNA sense strand sequence and modifications. TegChol=Teg linker + 3’-Cholesterol

Table S2. Human and mouse cross-targeting siRNA sequences and modification patterns.

*IFNGR1*_NM_000416_415	P(mU)#(fU)#(mU)(mC)(mU)(fU)(mU)(mU)(mU)(mG)(mU)(mC)(mC)#(fA)#(mA)#(fC)#(mC)#(mC)#(mU)#(fG)
	(mU)#(mU)#(mG)(mG)(fA)(fC)(fA)(mA)(fA)(mA)(mA)(mG)(mA)#(mA)#(mA)-TegChol
*IFNGR1*_NM_000416_416	P(mU)#(fA)#(mU)(mU)(mC)(fU)(mU)(mU)(mU)(mU)(mG)(mU)(mC)#(fC)#(mA)#(fA)#(mC)#(mC)#(mC)#(fU)
	(mU)#(mG)#(mG)(mA)(fC)(fA)(fA)(mA)(fA)(mA)(mG)(mA)(mA)#(mU)#(mA)-TegChol
*IFNGR1*_NM_000416_417	P(mU)#(fG)#(mA)(mU)(mU)(fC)(mU)(mU)(mU)(mU)(mU)(mG)(mU)#(fC)#(mC)#(fA)#(mA)#(mC)#(mC)#(fC)
	(mG)#(mG)#(mA)(mC)(fA)(fA)(fA)(mA)(fA)(mG)(mA)(mA)(mU)#(mC)#(mA)-TegChol
*IFNGR1*_NM_000416_418	P(mU)#(fA)#(mG)(mA)(mU)(fU)(mC)(mU)(mU)(mU)(mU)(mU)(mG)#(fU)#(mC)#(fC)#(mA)#(mA)#(mC)#(fC)
	(mG)#(mA)#(mC)(mA)(fA)(fA)(fA)(mA)(fG)(mA)(mA)(mU)(mC)#(mU)#(mA)-TegChol
*IFNGR1*_NM_000416_419	P(mU)#(fC)#(mA)(mG)(mA)(fU)(mU)(mC)(mU)(mU)(mU)(mU)(mU)#(fG)#(mU)#(fC)#(mC)#(mA)#(mA)#(fC)
	(mA)#(mC)#(mA)(mA)(fA)(fA)(fA)(mG)(fA)(mA)(mU)(mC)(mU)#(mG)#(mA)-TegChol
*IFNGR1*_NM_000416_987	P(mU)#(fU)#(mC)(mA)(mG)(fG)(mU)(mU)(mU)(mU)(mG)(mU)(mC)#(fU)#(mC)#(fU)#(mA)#(mA)#(mA)#(fG)
	(mG)#(mA)#(mG)(mA)(fC)(fA)(fA)(mA)(fA)(mC)(mC)(mU)(mG)#(mA)#(mA)-TegChol
*IFNGR1*_NM_000416_988	P(mU)#(fU)#(mU)(mC)(mA)(fG)(mG)(mU)(mU)(mU)(mU)(mG)(mU)#(fC)#(mU)#(fC)#(mU)#(mA)#(mA)#(fA)
	(mA)#(mG)#(mA)(mC)(fA)(fA)(fA)(mA)(fC)(mC)(mU)(mG)(mA)#(mA)#(mA)-TegChol
*IFNGR1*_NM_000416_989	P(mU)#(fA)#(mU)(mU)(mC)(fA)(mG)(mG)(mU)(mU)(mU)(mU)(mG)#(fU)#(mC)#(fU)#(mC)#(mU)#(mA)#(fA)
	(mG)#(mA)#(mC)(mA)(fA)(fA)(fA)(mC)(fC)(mU)(mG)(mA)(mA)#(mU)#(mA)-TegChol
*IFNGR1*_NM_000416_1244	P(mU)#(fA)#(mC)(mU)(mG)(fG)(mU)(mU)(mA)(mC)(mU)(mA)(mC)#(fU)#(mU)#(fA)#(mA)#(mA)#(mG)#(fG)
	(mA)#(mA)#(mG)(mU)(fA)(fG)(fU)(mA)(fA)(mC)(mC)(mA)(mG)#(mU)#(mA)-TegChol
*IFNGR1*_NM_000416_1245	P(mU)#(fG)#(mA)(mC)(mU)(fG)(mG)(mU)(mU)(mA)(mC)(mU)(mA)#(fC)#(mU)#(fU)#(mA)#(mA)#(mA)#(fG)
	(mA)#(mG)#(mU)(mA)(fG)(fU)(fA)(mA)(fC)(mC)(mA)(mG)(mU)#(mC)#(mA)-TegChol

Table S3. Mouse targeting siRNA sequences and modification patterns.

*Ifngr1*_NM_010511_306	P(mU)#(fG)#(mA)(mG)(mU)(fC)(mU)(mG)(mU)(mG)(mA)(mC)(mA)#(fU)#(mG)#(fU)#(mU)#(mC)#(mU)#(fG)
	(mC)#(mA)#(mU)(mG)(fU)(fC)(fA)(mC)(fA)(mG)(mA)(mC)(mU)#(mC)#(mA)-TegChol
*Ifngr1*_NM_010511_378	P(mU)#(fA)#(mA)(mU)(mG)(fA)(mU)(mC)(mA)(mG)(mA)(mA)(mA)#(fU)#(mG)#(fU)#(mU)#(mG)#(mG)#(fU)
	(mC)#(mA)#(mU)(mU)(fU)(fC)(fU)(mG)(fA)(mU)(mC)(mA)(mU)#(mU)#(mA)-TegChol
*Ifngr1*_NM_010511_804	P(mU)#(fA)#(mG)(mA)(mA)(fA)(mG)(mA)(mU)(mG)(mA)(mG)(mA)#(fU)#(mU)#(fC)#(mC)#(mG)#(mU)#(fC)
	(mA)#(mA)#(mU)(mC)(fU)(fC)(fA)(mU)(fC)(mU)(mU)(mU)(mC)#(mU)#(mA)-TegChol
*Ifngr1*_NM_010511_938	P(mU)#(fC)#(mC)(mA)(mA)(fU)(mA)(mC)(mG)(mC)(mA)(mA)(mA)#(fU)#(mA)#(fC)#(mC)#(mA)#(mG)#(fG)
	(mU)#(mA)#(mU)(mU)(fU)(fG)(fC)(mG)(fU)(mA)(mU)(mU)(mG)#(mG)#(mA)-TegChol
*Ifngr1*_NM_010511_947	P(mU)#(fC)#(mU)(mU)(mA)(fG)(mU)(mA)(mU)(mA)(mC)(mC)(mA)#(fA)#(mU)#(fA)#(mC)#(mG)#(mC)#(fA)
	(mA)#(mU)#(mU)(mG)(fG)(fU)(fA)(mU)(fA)(mC)(mU)(mA)(mA)#(mG)#(mA)-TegChol
*Ifngr1*_NM_010511_957	P(mU)#(fA)#(mU)(mG)(mA)(fA)(mU)(mU)(mC)(mU)(mU)(mC)(mU)#(fU)#(mA)#(fG)#(mU)#(mA)#(mU)#(fA)
	(mU)#(mA)#(mA)(mG)(fA)(fA)(fG)(mA)(fA)(mU)(mU)(mC)(mA)#(mU)#(mA)-TegChol
*Ifngr1*_NM_010511_1162	P(mU)#(fG)#(mA)(mA)(mA)(fG)(mU)(mU)(mC)(mU)(mU)(mC)(mC)#(fU)#(mG)#(fU)#(mU)#(mC)#(mU)#(fG)
	(mC)#(mA)#(mG)(mG)(fA)(fA)(fG)(mA)(fA)(mC)(mU)(mU)(mU)#(mC)#(mA)-TegChol
*Ifngr1*_NM_010511_1641	P(mU)#(fG)#(mU)(mU)(mA)(fG)(mU)(mA)(mU)(mU)(mA)(mG)(mC)#(fU)#(mA)#(fA)#(mU)#(mG)#(mU)#(fA)
	(mU)#(mA)#(mG)(mC)(fU)(fA)(fA)(mU)(fA)(mC)(mU)(mA)(mA)#(mC)#(mA)-TegChol
*Ifngr1*_NM_010511_1895	P(mU)#(fU)#(mG)(mA)(mA)(fC)(mU)(mU)(mA)(mC)(mA)(mU)(mA)#(fU)#(mA)#(fC)#(mA)#(mA)#(mA)#(fG)
	(mU)#(mA)#(mU)(mA)(fU)(fG)(fU)(mA)(fA)(mG)(mU)(mU)(mC)#(mA)#(mA)-TegChol
*Ifngr1*_NM_010511_1897	P(mU)#(fC)#(mA)(mU)(mG)(fA)(mA)(mC)(mU)(mU)(mA)(mC)(mA)#(fU)#(mA)#(fU)#(mA)#(mC)#(mA)#(fA)
	(mU)#(mA)#(mU)(mG)(fU)(fA)(fA)(mG)(fU)(mU)(mC)(mA)(mU)#(mG)#(mA)-TegChol
*Ifngr1*_NM_010511_1911	P(mU)#(fA)#(mA)(mA)(mC)(fA)(mU)(mA)(mU)(mU)(mA)(mU)(mA)#(fU)#(mA)#(fC)#(mA)#(mU)#(mG)#(fA)
	(mU)#(mA)#(mU)(mA)(fU)(fA)(fA)(mU)(fA)(mU)(mG)(mU)(mU)#(mU)#(mA)-TegChol
*Ifngr1*_NM_010511_2034	P(mU)#(fA)#(mA)(mA)(mG)(fU)(mA)(mU)(mG)(mU)(mA)(mC)(mA)#(fA)#(mG)#(fC)#(mU)#(mC)#(mC)#(fC)
	(mC)#(mU)#(mU)(mG)(fU)(fA)(fC)(mA)(fU)(mA)(mC)(mU)(mU)#(mU)#(mA)-TegChol

Table S4. Sequences and modification patterns of the two DCA-siRNA *Ifngr1* 1641 scaffolds used for *in vivo* studies.

Scaffold 1	V(mU)#(fG)#(mU)(mU)(mA)(fG)(mU)(mA)(mU)(mU)(mA)(mG)(mC)#(fU)#(mA)#(fA)#(mU)#(mG)#(mU)#(fA) [a]
	(mU)#(mA)#(mG)(mC)(fU)(fA)(fA)(mU)(fA)(mC)(mU)(mA)(mA)#(mC)#(mA)(dT)(dT)-DCA [b]
Scaffold 2 [c]	V(mU)#(fG)#(mU)(fU)(fA)(fG)(mU)(fA)(mU)(fU)(mA)(fG)(mC)(fU)#(mA)#(fA)#(mU)#(mG)#(mU)#(fA)#(mU)
	(mU)#(mU)#(mA)(fG)(mC)(fU)(mA)(fA)(mU)(fA)(mC)(mU)(mA)(fA)#(mC)#(mA)(dT)(dT)-DCA

[a] siRNA antisense strand sequence and modifications. m=2’-O-methyl; f=2’-Fluoro; #=Phosphorothioate; V=5’-Vinyl Phosphate; dT=Thymidine

[b] siRNA sense strand sequence and modifications. DCA=Docosanoic acid

[c] Scaffold 2 in a 21/16 nucleotides of antisense/sense configuration with balanced ratio of 2’-O-methyl to 2’-Fluoro may offer higher *in vivo* potency than scaffold 1 for certain siRNA sequences.

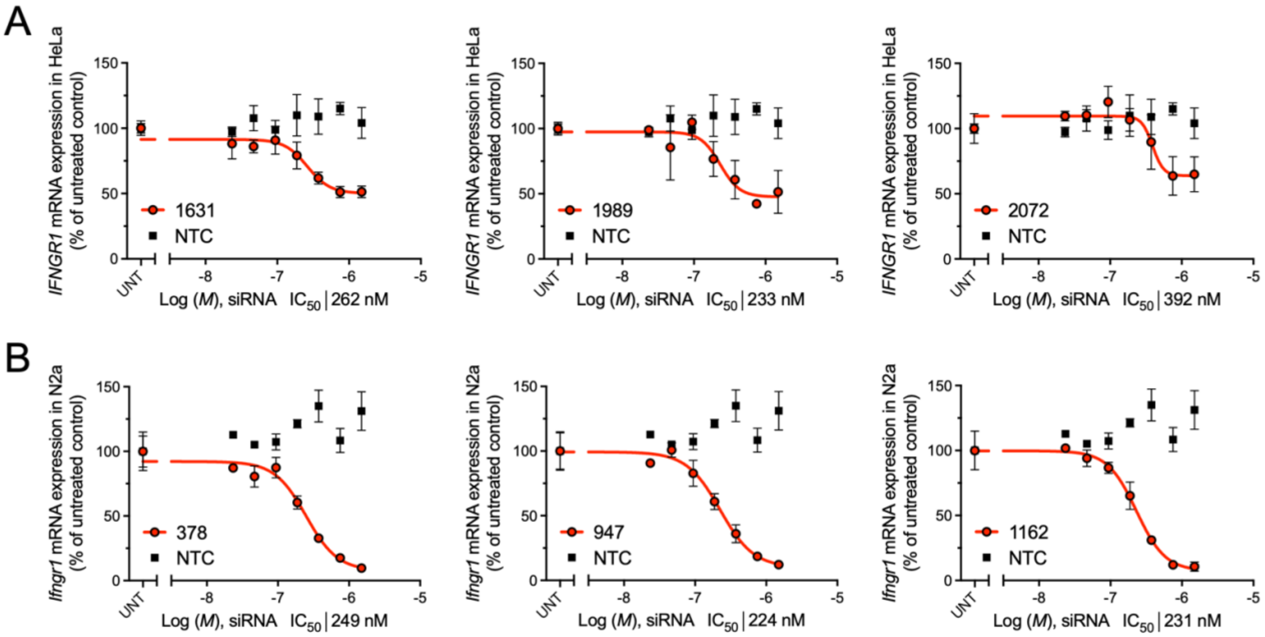

C

siRNA hits	Accession number	mRNA targeting region
Human *IFNGR1* 1631	NM_000416	3'-UTR, AAUUUCAUGAAAAGAUUAUG
Human *IFNGR1* 1989	NM_000416	3'-UTR, GCAAGCUUUUCAAAAUUGGA
Human *IFNGR1* 2072	NM_000416	3'-UTR, UAUGAAACAUUACAGUUAGA
Mouse *Ifngr1* 378	NM_010511	ORF, ACCAACAUUUCUGAUCAUUG
Mouse *Ifngr1* 947	NM_010511	ORF, UGCGUAUUGGUAUACUAAGA
Mouse *Ifngr1* 1162	NM_010511	ORF, CAGAACAGGAAGAACUUUCA

Figure S1. Dose response of top hits in silencing human *IFNGR1* and mouse *Ifngr1*. (A) Human *IFNGR1* silencing in HeLa cells. (B) Mouse *Ifngr1* silencing in N2a cells. 7-point dose response curve generated by treating cells with fully modified cholesterol-conjugated siRNAs at 1.5 μM with progressive 2-fold serial dilutions for 72 h (n=3, mean ± SD). *M* represents the molar concentration of siRNA (n=3, mean ± SD). (C) Targeting regions of top siRNA hits in human *IFNGR1* and mouse *Ifngr1* mRNA transcripts.

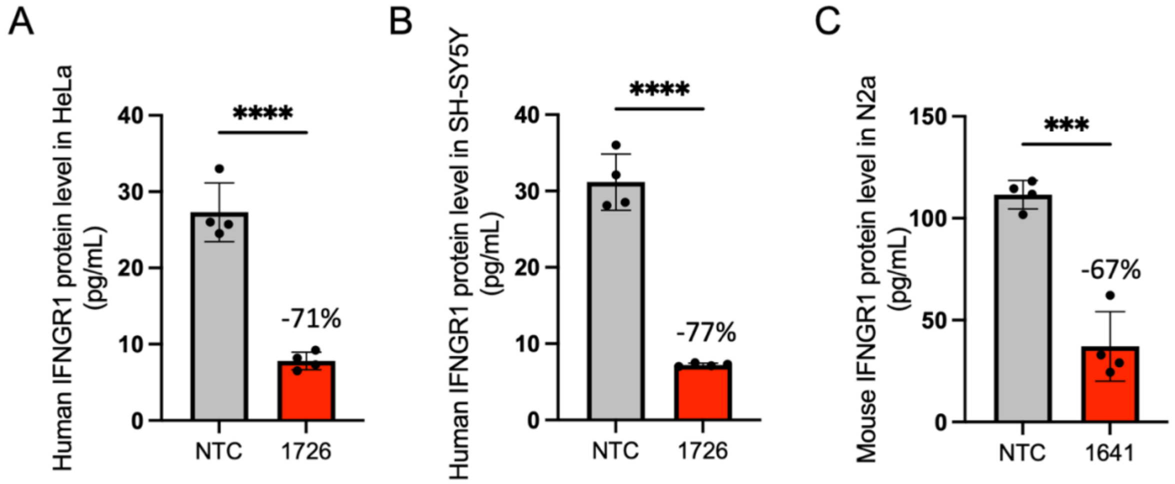

Figure S2. IFNGR1 protein expressions in human HeLa, SH-SY5Y, and mouse N2a cells. Human lead compound 1726 reduces IFNGR1 expression in (A) human HeLa cells, and (B) SH-SY5Y cells. (C) Mouse lead compound 1641 reduces IFNGR1 expression in mouse N2a cells. Cells were treated with fully modified cholesterol-conjugated siRNAs at 1.5 μM for 72 h (n=4, mean ± SD). Protein expressions were determined by ELISA and normalized to total protein levels (quantified by Bradford assays). Data are represented as mean ± SD and analyzed by unpaired t test (***p<0.001, ****p<0.0001).

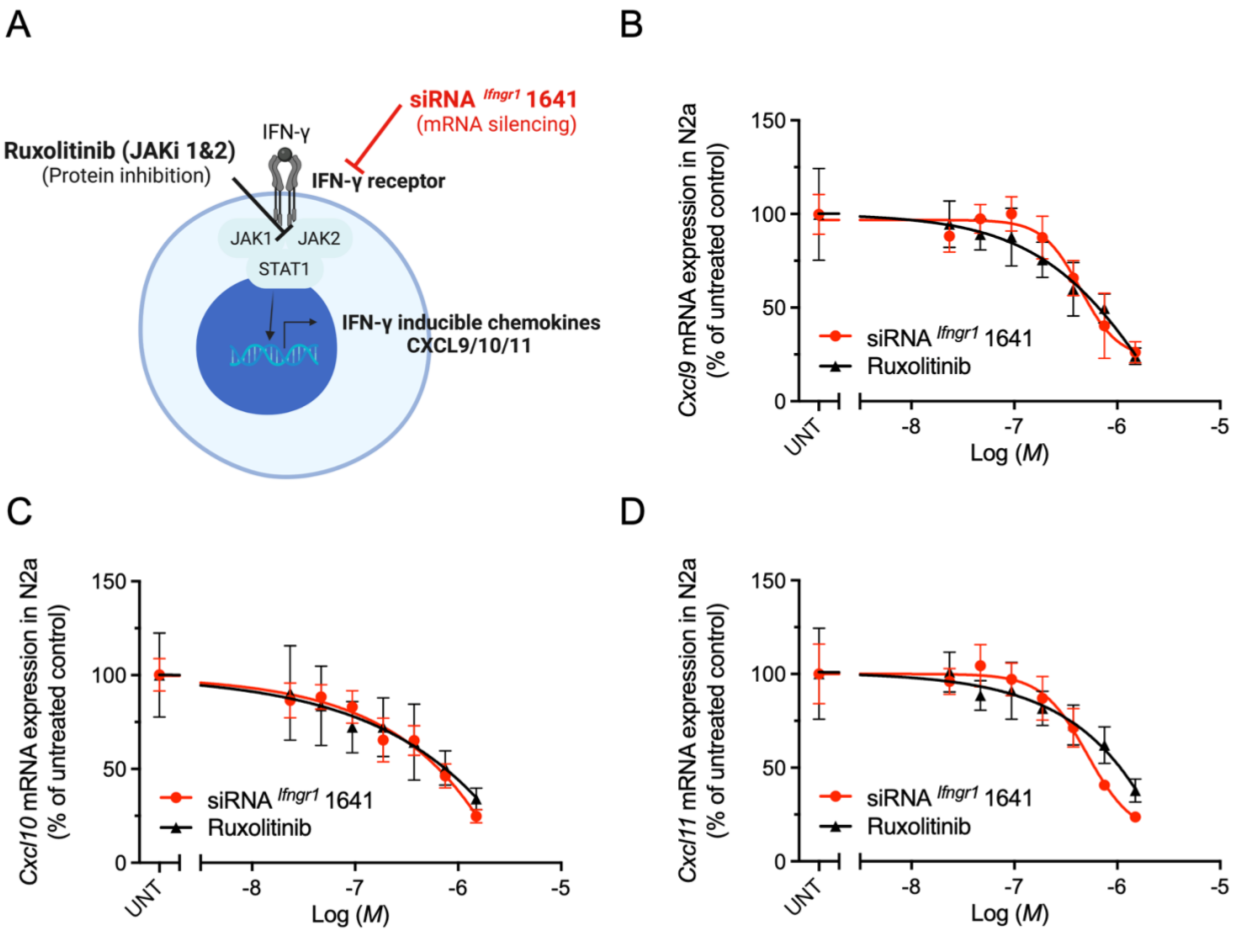

Figure S3. Comparison of siRNA *Ifngr1* 1641 to Ruxolitinib in inhibiting IFN-γ signaling. (A) siRNA *Ifngr1* 1641 and small molecule JAK1&2 inhibitor Ruxolitinib inhibit IFN-γ signaling in different mechanisms. Mouse N2a cells were treated with cholesterol-conjugated siRNA *Ifngr1* 1641 or Ruxolitinib at 1.5 μM with progressive 2-fold serial dilutions for 72 h prior to IFN-γ signaling stimulation. (B), (C), and (D) 7-point dose response curves of *Cxcl9*, *Cxcl10*, and *Cxcl11* mRNA expression at 6 h post signal stimulation (QuantiGene 2.0 assay). M represents the molar concentration of siRNA or Ruxolitinib (n=6, mean ± SD).

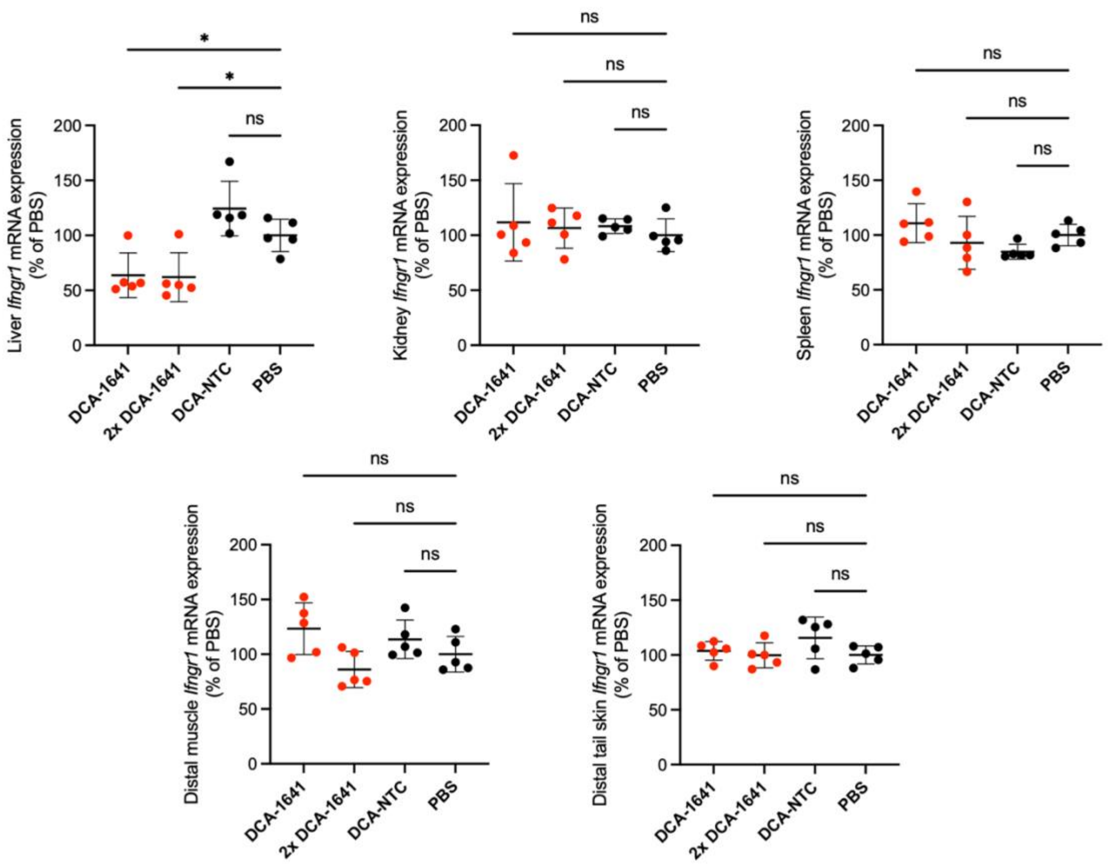

Figure S4. Systemic silencing of *Ifngr1* in liver, kidney, spleen, distal muscle, and distal tail skin. Mice were injected subcutaneously (between shoulders) with a single dose or two doses (2x, 24 h apart; n=5) of DCA-siRNA ^{Ifngr1} 1641. Tissues were collected at 1 week post-injection and *Ifngr1* mRNA levels were measured by QuantiGene 2.0 assays. Data are represented as percent of PBS control (mean ± SD) and analyzed by ordinary one-way ANOVA (*p<0.05; ns, not significant).

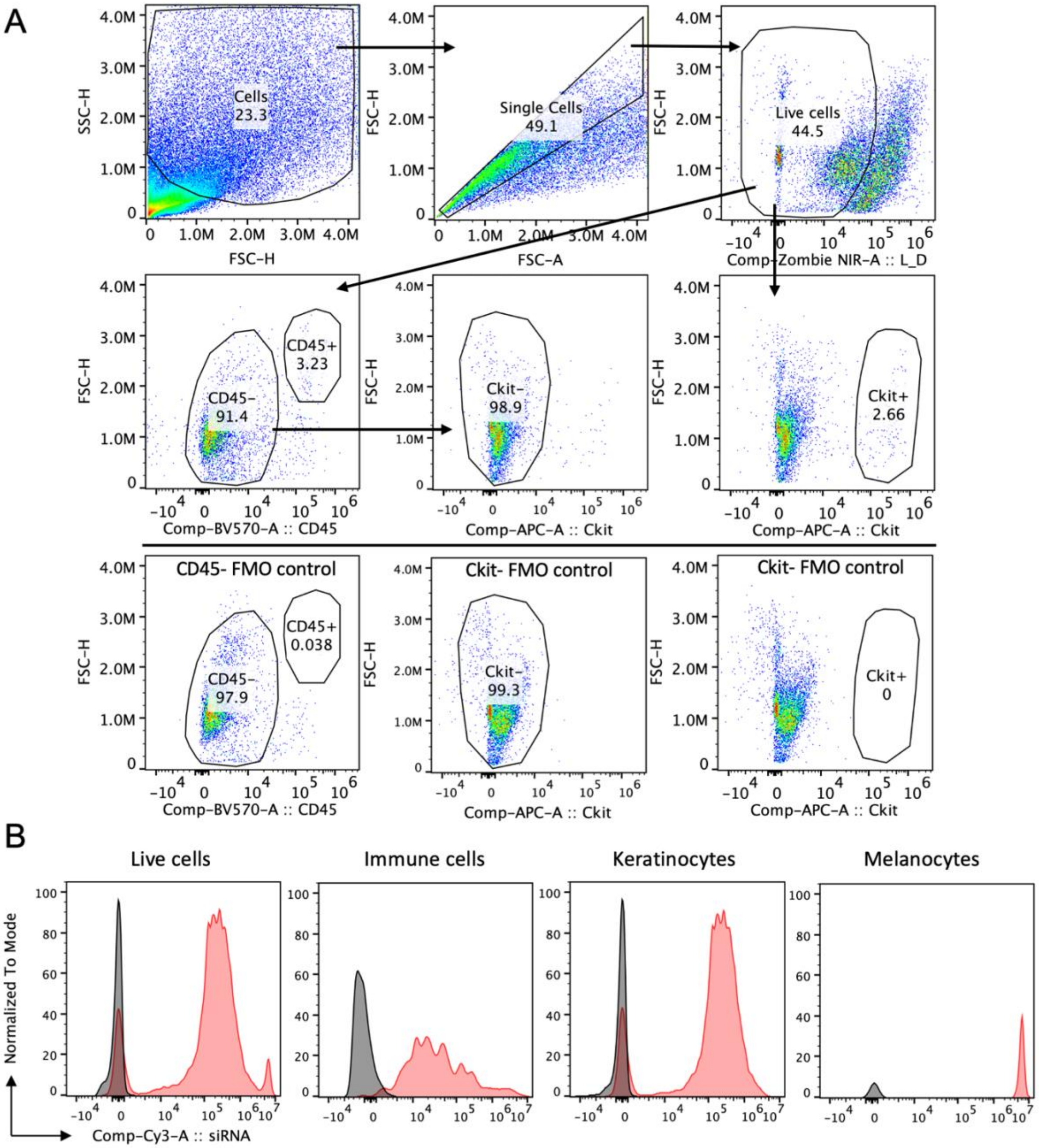

Figure S5. Cy3-DCA-siRNA $^{\textit{Ifngr1}}$ 1641 delivery efficiency to major cell types of the epidermis. Epidermal single cell suspensions were analyzed by fluorescence flow cytometry with CD45 and Ckit markers to determine the delivery efficiency of siRNA into major epidermal cell types. (A) Gating strategy: single cells were gated by FSC-H/SSC-H followed by FSC-A/FSC-H exclusion. Dead cells were excluded by Zombie NIR staining. Immune cells and melanocytes were separated by CD45^{+} and Ckit^{+} expression, respectively. Keratinocytes constitute the most cells in CD45^{-} and Ckit^{-} double negative population, thus no further staining was performed. Controls of CD45^{-} and Ckit^{-} fluorescence minus one (FMO) were used for assisting negative gating. (B) Representative histograms of Cy3-labeled siRNA delivery efficiency to epidermal cells (overall), and in epidermal immune cells, keratinocytes, and melanocytes. Red peaks: Cy3 fluorescence intensity in cells of Cy3-siRNA treated mice; Gray peaks: PBS control.

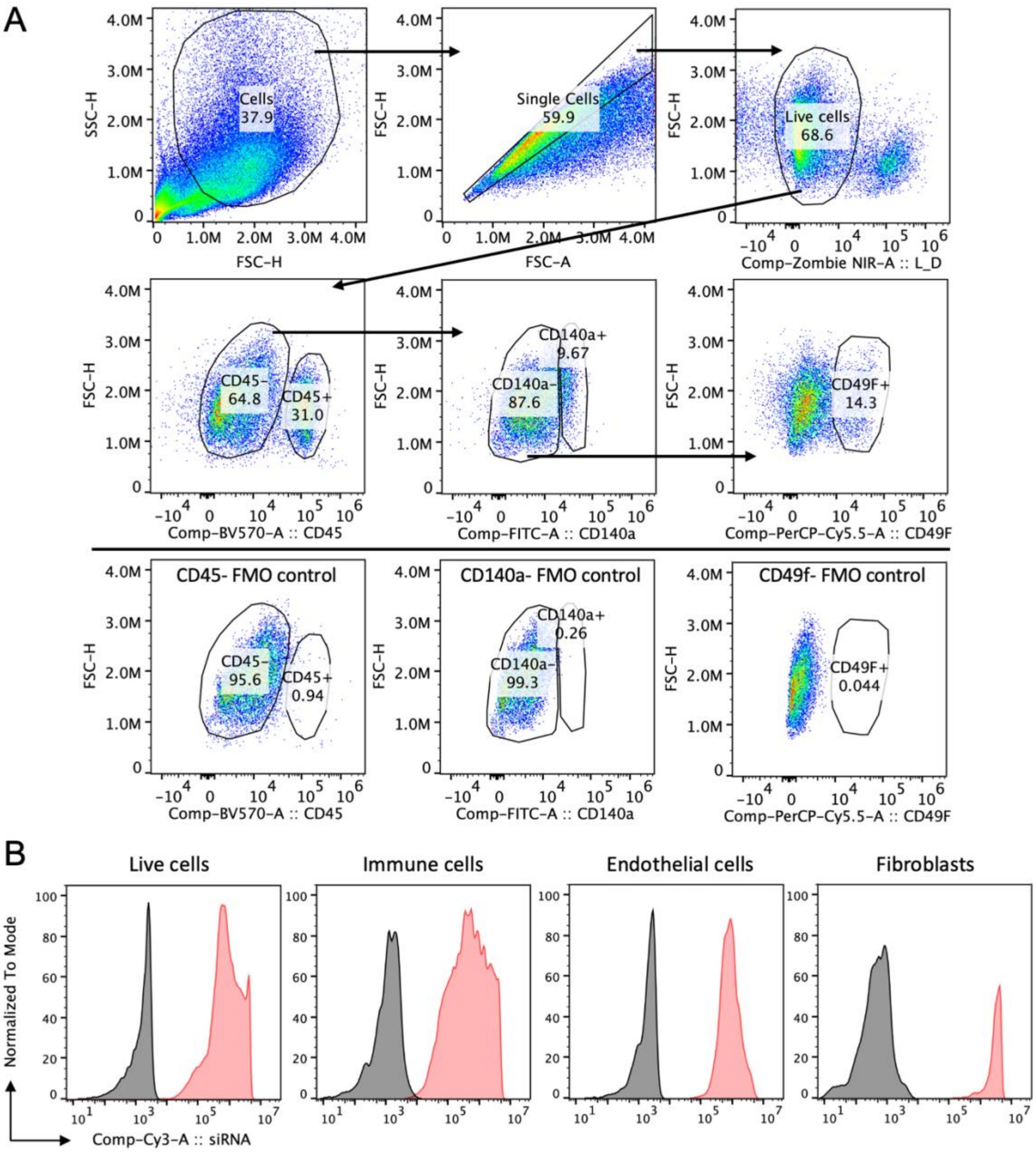

Figure S6. Cy3-DCA-siRNA $^{\mathit{Ifngr1}}$ 1641 delivery in major cell types. Dermal single cell suspensions were analyzed by fluorescence flow cytometry with CD45, CD140a, and CD49f markers to determine the delivery efficiency of siRNA into major dermal cell types. (A) Gating strategy: single cells were gated by FSC-H/SSC-H followed by FSC-A/FSC-H exclusion. Dead cells were excluded by Zombie NIR staining. Immune cells were gated by CD45^{+} expression. Fibroblasts were gated by CD45^{-} and CD140a^{+} expression. Endothelial cells were gated by CD45^{-}, CD140a^{-}, and CD49f^{+} expression. Controls of CD45^{-}, CD140a-, and CD49f- fluorescence minus one (FMO) were used for assisting negative gating. (B) Representative histograms of Cy3-labeled siRNA delivery efficiency to dermal cells (overall), and to dermal immune cells, fibroblast, and endothelial cells. Red peaks: Cy3 fluorescence intensity in cells of Cy3-siRNA treated mice; Gray peaks: PBS control.

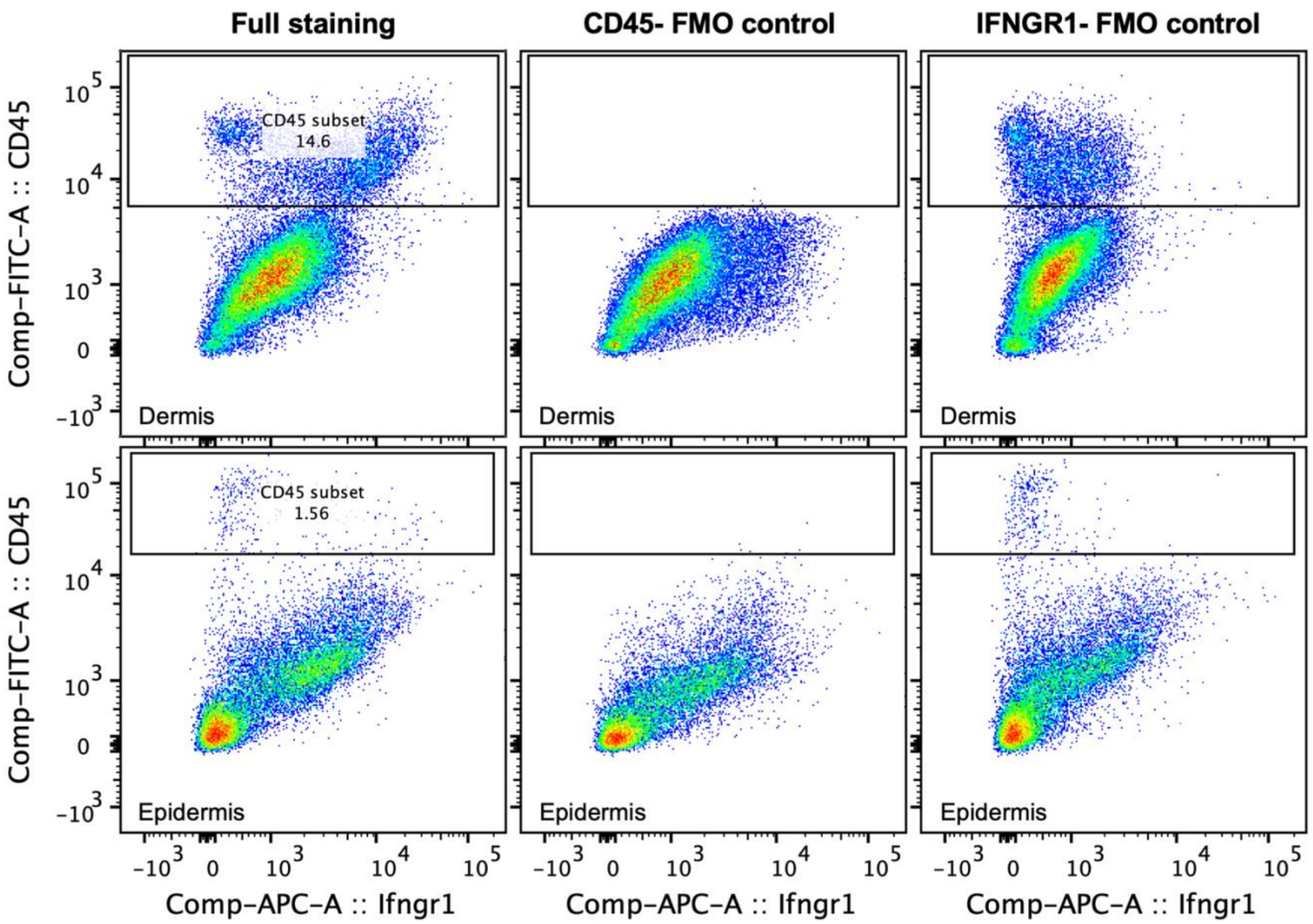

Figure S7. IFNGR1 expression ratio is higher in CD45^{+} hematopoietic cells of dermis than in epidermis. Representative graphs show that CD45 and IFNGR1 staining in dermis and epidermis with individual fluorescence minus one (FMO) Ab negative controls. CD45^{-} and IFNGR1^{-} FMO controls were used for assisting negative gating. The expression of IFNGR1 in epidermis is relatively low compared to dermis such that a reliable quantification of cell population is limited in the present analysis. To evaluate the treatment effect of DCA-siRNA ^{Ifngr1} 1641 in dermal cells, the CD45^{+} cells were gated as in whole as the population may contain multiple sub-populations expressing IFNGR1 to varying extent.
